# Amiloride ameliorates muscle wasting in cancer cachexia through inhibiting tumor-derived exosome release

**DOI:** 10.1186/s13395-021-00274-5

**Published:** 2021-07-06

**Authors:** Lin Zhou, Tong Zhang, Wei Shao, Ruohan Lu, Lin Wang, Haisheng Liu, Bin Jiang, Shiqin Li, Huiqin Zhuo, Suheng Wang, Qinxi Li, Caihua Huang, Donghai Lin

**Affiliations:** 1 Key Laboratory for Chemical Biology of Fujian Province, MOE Key Laboratory of Spectrochemical Analysis & Instrumentation, College of Chemistry and Chemical Engineering, Xiamen University, Xiamen, 361005 China; 2 Xiamen Cardiovascular Hospital, Xiamen University, Xiamen, 361000 China; 3 Department of Oncology, Institute of Gastrointestinal Oncology, Zhongshan Hospital, Xiamen University, Xiamen, 361004 China; 4 State Key Laboratory of Cellular Stress Biology, School of Life Sciences, Xiamen University, Xiamen, 361102 China; 5 Department of Medical Oncology, Xiang’an Hospital of Xiamen University, Xiamen, China; 6 Department of Gastrointestinal Surgery, The Affiliated Zhongshan Hospital, Xiamen University, Xiamen, 361004 Fujian China; 7 Collaborative Innovation Center of Chemistry for Energy Materials, College of Chemistry and Chemical Engineering, Xiamen University, Xiamen, 361005 China; 8 Research and Communication Center of Exercise and Health, Xiamen University of Technology, Xiamen, 361024 China; 9 High-field NMR Center, College of Chemistry and Chemical Engineering, Xiamen University, Xiamen, 361005 China

**Keywords:** Amiloride, Cancer cachexia, Muscle wasting, Exosome, Exosome-release inhibition

## Abstract

**Background:**

Cancer cachexia (CAC) reduces patient survival and quality of life. Developments of efficient therapeutic strategies are required for the CAC treatments. This long-term process could be shortened by the drug-repositioning approach which exploits old drugs approved for non-cachexia disease. Amiloride, a diuretic drug, is clinically used for treatments of hypertension and edema due to heart failure. Here, we explored the effects of the amiloride treatment for ameliorating muscle wasting in murine models of cancer cachexia.

**Methods:**

The CT26 and LLC tumor cells were subcutaneously injected into mice to induce colon cancer cachexia and lung cancer cachexia, respectively. Amiloride was intraperitoneally injected daily once tumors were formed. Cachexia features of the CT26 model and the LLC model were separately characterized by phenotypic, histopathologic and biochemical analyses. Plasma exosomes and muscle atrophy-related proteins were quantitatively analyzed. Integrative NMR-based metabolomic and transcriptomic analyses were conducted to identify significantly altered metabolic pathways and distinctly changed metabolism-related biological processes in gastrocnemius.

**Results:**

The CT26 and LLC cachexia models displayed prominent cachexia features including decreases in body weight, skeletal muscle, adipose tissue, and muscle strength. The amiloride treatment in tumor-bearing mice distinctly alleviated muscle atrophy and relieved cachexia-related features without affecting tumor growth. Both the CT26 and LLC cachexia mice showed increased plasma exosome densities which were largely derived from tumors. Significantly, the amiloride treatment inhibited tumor-derived exosome release, which did not obviously affect exosome secretion from non-neoplastic tissues or induce observable systemic toxicities in normal healthy mice. Integrative-omics revealed significant metabolic impairments in cachectic gastrocnemius, including promoted muscular catabolism, inhibited muscular protein synthesis, blocked glycolysis, and impeded ketone body oxidation. The amiloride treatment evidently improved the metabolic impairments in cachectic gastrocnemius.

**Conclusions:**

Amiloride ameliorates cachectic muscle wasting and alleviates cancer cachexia progression through inhibiting tumor-derived exosome release. Our results are beneficial to understanding the underlying molecular mechanisms, shedding light on the potentials of amiloride in cachexia therapy.

**Supplementary Information:**

The online version contains supplementary material available at 10.1186/s13395-021-00274-5.

## Introduction

Cachexia is a systemic metabolic syndrome defined by involuntary body weight and skeletal muscle loss (with or without fat loss) and cannot be fully reversed by conventional nutritional supplementations [1]. Driven by a complicated combination of endocrine and metabolic disorders as well as central nervous system perturbations, cachexia is characterized by several predominant features including reduced food intake, decreased muscle mass, excess catabolism, unnecessary energy expenditure, and hyper-inflammatory response [2]. Pathologic mechanisms of cancer cachexia are closely related to activation of proteolysis, autophagy, lipolysis, and inflammation [3].

Cachexia is usually associated with chronic and malignant diseases, including kidney disease, heart failure, neurological disease, chronic obstructive pulmonary disease, AIDS, and, especially, cancer [4]. As one of the leading causes of death worldwide, cancer accounts for an estimated 9.6 million deaths in 2018, and nearly 80% of cancer patients are affected by cachexia in different grades [5]. Cancer cachexia (CAC) directly causes about one third of fatalities in cancer patients with significant skeletal muscle wasting [6]. Efficient therapeutic strategies for the CAC treatments are urgently needed [4]. Recently, mounting researches indicated that tumor-derived exosomes contribute to cancer cachexia through mediating the cross-talk between tumors and distally located skeletal muscles, resulting in decreased muscle weight, impaired organismal function, suppressed therapeutical response, and reduced quality of life, as well as remarkably enhanced cancer-related mortality [7–10]. These works might provide a new strategy for the CAC treatments based on inhibition of tumor-derived exosomes release.

Amiloride is an old drug with potassium-sparing diuretic function (upon inhibition of Na^+^/H^+^ and Na^+^/K^+^ exchangers), which has been clinically employed in the treatments of hypertension, hypokalemia, edema, and congestive heart failure for decades [11]. Most importantly, amiloride can inhibit exosome release from cells and reverse exosome-promoted pathogenic processes, including autoimmune disease and immuno-suppressive regulation [12, 13]. However, no attempts have been reported to ameliorate cachectic muscle wasting through inhibiting tumor-derived exosome release. Given that tumor-derived exosomes are implicated in mediating cachectic muscle wasting, we speculated that amiloride might have some effects for ameliorating muscle wasting in cancer cachexia.

In the present work, we sought to determine whether amiloride was able to ameliorate muscle wasting in murine cachexia models. Furthermore, we addressed molecular mechanisms of tumor-derived exosomes promoting muscle wasting by integrative metabolomic and transcriptomic analyses, which provides the mechanistic rationale for exploiting clinical potentials of amiloride for improving the CAC treatments. Our results demonstrated that the amiloride treatment could significantly ameliorate muscle wasting in cancer cachexia and thus alleviate the CAC progression through inhibiting tumor-derived exosome release.

## Methods

### Patient blood sample collection

Patient blood samples were firstly kept in anti-coagulation (100 mM sodium citrate) tubes and then centrifuged (1000*g*, 10 min, 4 °C) to obtain platelet-free plasma within 2 h after blood collection. The plasma was aliquoted and collected in cryovials and kept at − 80 °C until used.

### Exosome isolation and characterization

Exosomes in either patient/mouse plasma or culture media of CT26/LLC cells were isolated by ultracentrifugation [14, 15]. A Beckman Coulter XE-90K Ultracentrifuge equipped with an SW 41 Ti rotor was used for the ultracentrifugation. Particle sizes of exosomes were analyzed using ZetaView® Nanoparticle Tracking Analyzer (Particle Metrix GmbH, Meerbusch, Germany) and FEI Tecnai 20 transmission electron microscope (Thermo Fisher, USA) according to the manufacturer’s manual [16] or the published protocol [17], respectively.

### Exosome density and purity measurements

Exosome density and purity were analyzed with a two-channel high-sensitivity nano flow cytometer (HSFCM) according to the protocol described previously [15]. The HSFCM was developed by the Laboratory of Professor XM Yan from College of Chemistry and Chemical Engineering, Xiamen University, which has been commercialized by NanoFCM Inc, China. To assess exosome purities, 1% final volume of Triton X-100 (Sigma-Aldrich, USA) was added to exosome suspensions, and the HSFCM measurement was repeated after incubation of 30 min on ice.

### Cell culture

The LLC, CT26, and HEK 293T cells were purchased from the China Center for Typical Culture Collection (CCTCC). C2C12 cells were provided by Stem Cell Bank, Chinese Academy of Sciences. LLC and CT26 cells were cultured in DMEM and RPMI-1640, respectively. Both culture media were supplemented with 100 units/mL penicillin, 100 μg/mL streptomycin, and 10% fetal bovine serum (Hyclone, USA). All cells were cultured in a humidified atmosphere of 5% CO_2_ at 37 °C. Culture media of LLC and CT26 cells were collected and centrifuged (1000*g*, 5 min, 4 °C) after 48 h of culture. C2C12 myoblasts were cultured to 85–90% confluence in DMEM growth medium. Myoblast differentiation was induced by incubation for 96 h in DMEM supplemented with 2% heat-inactivated horse serum. C2C12 myoblasts were used within ten generations of culture.

### Lentiviral expression of shRNA in tumor cells

The pLKO.1-puro lentivirus vector was used to express the shRNAs. The virus was generated by four co-transfected plasmids, including the lentiviral vector, pMDLg/pRRE, pRSV-Rev, and pMD2 in HEK 293T cells. At 48 h, virus-containing supernatants were collected for transduction in CT26 and LLC cells. The shRNAs against mouse *Rab27a* and *Rab27b* were 5′-GCTTCTGTTCGACCTGACAAA-3′ and 5′-GCTTCTGGACTTAATCATGAA-3′, respectively.

### Animal experiments

To assess the effects of amiloride for ameliorating muscle atrophy in vivo, we constructed murine models of CT26 (colon) and LLC (lung) cancer cachexia (Fig. S1). Adult (age 6–8 weeks) C57BL/6 and BALB/c male mice were purchased from Shanghai SLAC Laboratory Animal Co., Ltd. Mice were individually housed, acclimated to their cages and human handling for 1 week before animal experiments, and maintained in conditions of constant temperature and 12-h light/12-h dark cycles.

Tumor cells were subcutaneously injected into the right flank of mice on day 0. In detail, BALB/c mice were inoculated with the CT26 cells (1.0 × 10^6^/100 μL) to induce colon cancer cachexia, while C57BL/6 mice were inoculated with the LLC cells (7 × 10^5^/100 μL) to induce lung cancer cachexia. Both BALB/c and C57BL/6 mice showed palpable tumors (about 5 mm in diameter) on day 9 after the inoculation. Both CT26-bearing mice and LLC-bearing mice were randomly divided into 2 groups (*n* = 8 per group): one group of mice intraperitoneally injected daily with PBS from day 9 (CAC mice), another group of mice intraperitoneally injected daily with amiloride dissolved in PBS at a dose of 2 mg/kg (AM mice). Furthermore, The *Rab27* knock-down tumor cells were subcutaneously injected into the mice following the same procedure (KD mice). Similarly, normal control mice were injected with PBS on day 0 (NOR mice). Both the KD and NOR mice were intraperitoneally injected with PBS daily from day 9.

Mouse body weights were monitored every 3-day post-tumor implantation, and food intakes were measured every day. Tumor volumes were calculated every 3-day post-tumor implantation using the formula: tumor volume (mm^3^) = 0.52 × length × width^2^, in which the length and perpendicular width were measured with a vernier caliper. Forelimb grip forces were measured with a Grip Strength Meter (YLS-13A, Shandong Academy of Medical Sciences, China). For each mouse, the grip strength was defined as the average of five successive measurements. On day 30, the mice were sacrificed. Both tumors and gastrocnemius were removed, weighed, and quickly frozen in liquid nitrogen for subsequent analysis. The mouse blood samples were firstly kept in anti-coagulation (100 mM sodium citrate) tubes and pro-coagulation tubes and then centrifuged (1000*g*, 10 min, 4 °C) to obtain platelet-free plasma and serum, respectively, within 2 h after blood collection. The plasma exosomes were quantitatively analyzed. Serum levels of TNF-α, IL-6, and IL-1β were measured by ELISA kit (R&D Systems China) according to the manufacturer’s instructions.

### Muscular toxicity evaluation

We evaluated potential muscular toxicities of the amiloride treatment in the CT26 and LLC models using C57BL/6 and BALB/c normal control mice, respectively. Either 12 C57BL/6 mice or 12 BALB/c mice were divided into 2 groups: NOR mice and NOR-AM mice, 6 per group. PBS was subcutaneously injected into the right flank of the NOR and NOR-AM mice on day 0. From day 9, the NOR-AM mice were intraperitoneally injected daily with amiloride at the same dose of 2 mg/kg following the procedure described above, whereas the NOR mice with PBS continually. Both the NOR-AM and the NOR mice were sacrificed on day 30, and toxic effects of amiloride were assessed by the differences in body weight, gastrocnemius muscle weight, and plasma exosome density between the NOR-AM mice and the NOR mice.

### Histology study

C2C12 myotubes were fixed with pre-cold methanol for 30 s, stained with 0.1% crystal violet solution for 10 min, and rinsed with distilled water before taking microscopic photographs. Myotube diameters were measured in a total of 200 myotubes from ≥ 10 random fields. Mouse gastrocnemius was collected and fixed in 4% PFA. Paraffin sections were stained with H&E, and stained slides were assessed using phase-contrast microscopy. Myofiber areas were quantified by using ImageJ. To produce frequency distribution histograms, five view fields were measured with about 200 myofibers per field in each section.

### Protein expression analysis

Proteins were extracted using RIPA lysis buffer containing protease inhibitor and phosphorylation protease inhibitor cocktails (Thermo Fisher, USA). The homogenates were then sonicated for 35 s and centrifuged (11,000*g*, 10 min, 4 °C) to remove the debris. The supernatants were collected, and protein concentrations were determined by BCA Protein Assay Kit (Beyotime Biotechnology). Then, the denatured samples were subjected to SDS-PAGE and transferred to PVDF membranes (GE Healthcare, USA) for immunoblotting analysis. After blocking with 5% non-fat milk in Tris-buffered saline containing 0.1% Tween 20 for 1 h, the membranes were probed with corresponding antibodies. Proteins were visualized by enhanced chemiluminescence using horseradish peroxidase-conjugated antibodies and band densities were quantified by the ImageJ software.

### NMR-based metabolomic analysis

Aqueous metabolites were extracted from mouse gastrocnemius for NMR-based metabolomic analysis according to the protocol described previously [18–20]. All NMR experiments were performed at 25 °C on a Bruker Avance III 850 MHz spectrometer (Bruker BioSpin, Germany) equipped with a TCI cryoprobe. Both the unsupervised principal component analysis (PCA) and supervised partial least-squares discriminant analysis (PLS-DA) were applied to compare metabolic profiles of gastrocnemius among the four groups of the NOR, CAC, AM, and KD mice by using the SIMCA 14.1 software (MKS Umetrics AB, Sweden). The metabolic pathway analysis was performed to identify significantly altered metabolic pathways (significant pathways) on the MetaboAnalyst 4.0 webserver (https://www.metaboanalyst.ca). This webserver was also used to obtain heat-map plots of relative metabolite levels, and the Pearson’s correlation coefficients between catabolic protein expressions and metabolite levels.

### Transcriptomic analysis

The RNA-seq experiments were performed by Gene Denovo Biotechnology Co. (Guangzhou, China). The NOISeq R/Bioc package was used to identify differentially expressed genes (DEGs) from pairwise comparisons among the groups of mouse gastrocnemius with two criteria: fold change (FC) ≥ 1.5 or FC ≤ 0.67, false discovery rate (FDR) ≤ 0.05. The Kyoto Encyclopedia of Genes and Genomes (KEGG) database was employed to conduct the pathway enrichment analysis based on the identified DEGs. Pathways with Q value ≤ 0.01 were identified to be significantly changed biological processes.

### General statistical analysis

Experimental data were expressed as means ± SD. For the quantitative comparison between two groups, data were analyzed by Student’s t test analysis using GraphPad Prism. For pairwise comparisons among three or more groups, data were analyzed by using one-way ANOVA followed by Tukey’s multiple comparison test using the SPSS 19 software. Statistical significances were as follows: *p* > 0.05 (NS), *p* < 0.05 (*), p < 0.01 (**), *p* < 0.001 (***), and *p* < 0.0001 (****).

### Antibodies and reagents

The detailed information about antibodies and reagents is displayed in Table S1.

## Results

### Amiloride treatment ameliorated cachectic muscle atrophy in mice

**Fig. 1:**
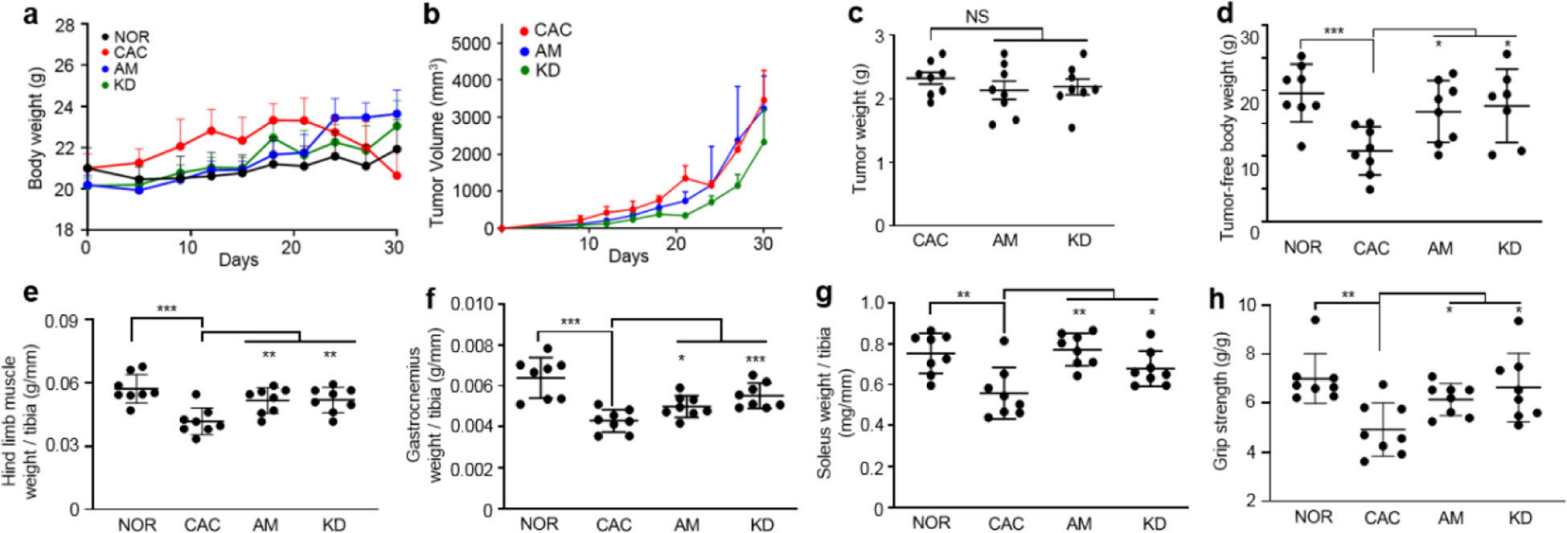
Amiloride alleviated cachexia progression in the CT26 murine model. **a**, **b** Body weight and tumor volume growth curves of the mice during the processes of establishing the animal models. **c**–**h** Characterization of cachexia features in the mice on day 30 ( = 6-8). **c**, **d** Tumor weights and tumor-free body weights of the mice. **e** Hindlimb muscle weights normalized to tibia length. **f** Gastrocnemius weights normalized to tibia length. **g** Soleus weights normalized to tibia length. **h** Grip strengths normalized to bodyweight. Statistical significances: *p* > 0.05, NS; *p* < 0.05, *; *p* < 0.01, **; *p* < 0.001, ***. NOR, C57BL/6 or BALB/c normal control mice; CAC, CT26 cachexia mice; AM, amiloride-treated mice; KD, mice inoculated with *Rab27*-knockdown CT26 cells

**Fig. 2:**
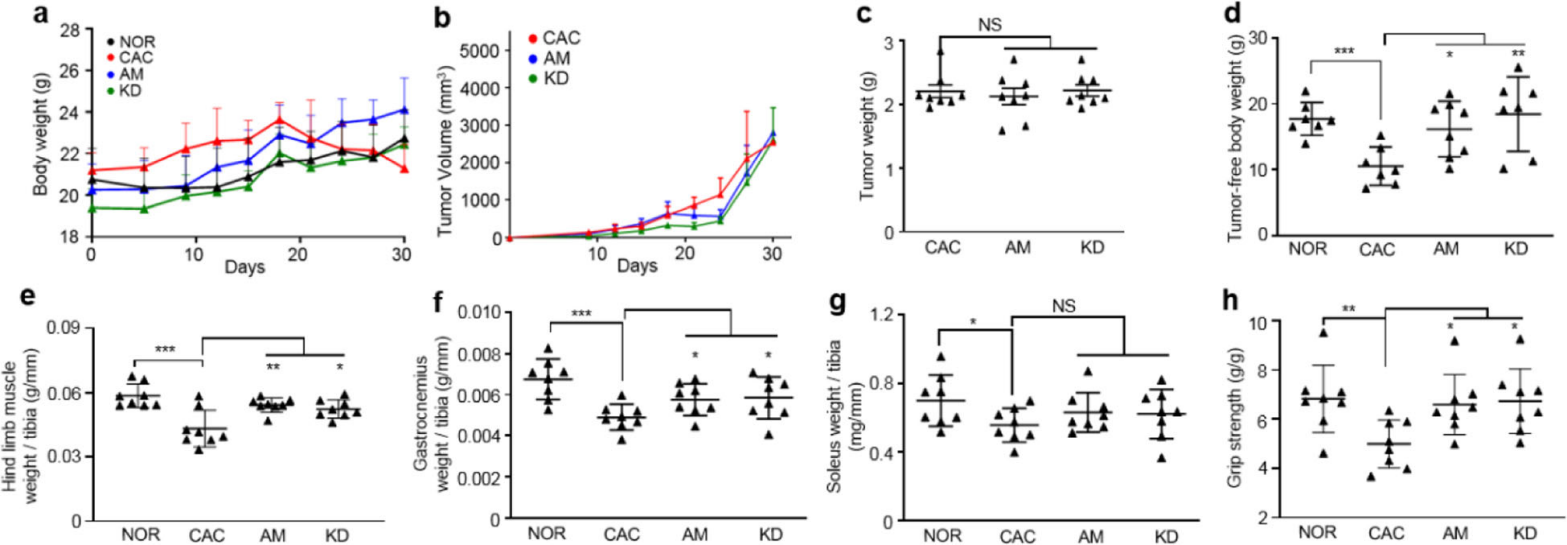
Amiloride alleviated cachexia progression in the LLC murine model. **a**, **b** Body weight and tumor volume growth curves of the mice during the processes of establishing the animal models. **c**–**h** Characterization of cachexia features in the mice on day 30 (n = 6-8). **c**, **d** Tumor weights and tumor-free body weights of the mice. **e** Hindlimb muscle weights normalized to tibia length. **f** Gastrocnemius weights normalized to tibia length. **g** Soleus weights normalized to tibia length. **h** Grip strengths normalized to bodyweight. Statistical significances: *p* > 0.05, NS; *p* < 0.05, *; *p* < 0.01, **; *p* < 0.001, ***. NOR, C57BL/6 or BALB/c normal control mice; CAC, LLC cachexia mice; AM, amiloride-treated mice; KD, mice inoculated with *Rab27*-knockdown LLC cells

**Fig. 3:**
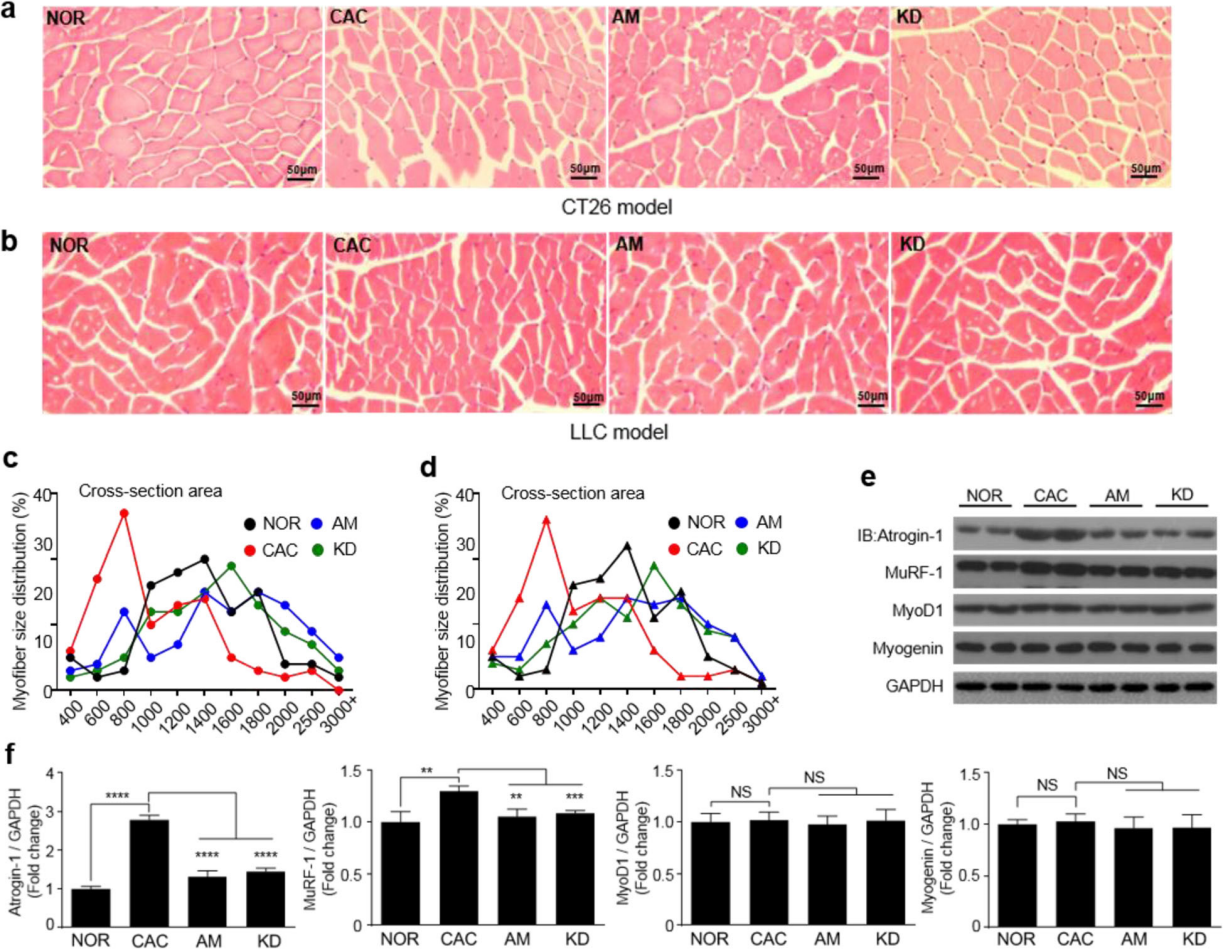
Amiloride profoundly ameliorated gastrocnemius atrophy in the CT26 model and the LLC model. Cancer cachexia features of gastrocnemius were analyzed for the NOR, CAC, AM, and KD mice on day 30. **a**, **b** Representative microscope pictures of hematoxylin and eosin staining for gastrocnemius of the CT26 model (**a**) and the LLC model (**b**) (scale bar = 50 μm). **c**, **d** Myofiber size distributions of gastrocnemius of the CT26 model (**c**) and the LLC model (**d**) (n = 5, 200 myofibers were used). **e** Expressions of MuRF-1, Atrogin-1, MyoD1, and Myogenin proteins in gastrocnemius of the CT26 and LLC models. **f** Quantification of the expressed proteins (n = 4). Statistical significances: *p* > 0.05, NS; *p* < 0.05, *; *p* < 0.01, **; *p* < 0.001, ***; *p* < 0.0001, ****. NOR, C57BL/6 or BALB/c normal control mice; CAC, CT26/LLC cachexia mice; AM, amiloride-treated mice; KD, mice inoculated with Rab27-knockdown CT26/LLC cells

The processes of establishing mouse models are illustrated in Fig. S1. The CT26 and LLC cells or PBS were subcutaneously injected into the mice on day 0, while amiloride or PBS was intraperitoneally injected daily from day 9 once mice developed palpable tumors. Both mouse body weights and tumor volumes were monitored along the process of establishing the CT26 cachexia model or the LLC cachexia model (Fig. 1a, b; Fig. 2a, b). On day 30 after tumor inoculation, the tumor-bearing mice displayed significant CAC features including decreases in body weights (mean loss rate: CT26, 5.9%; LLC, 6.5%), tumor-free body weights (TFBWs, mean loss rate: CT26, 14.8%; LLC, 12.4%), hind limb muscle weights (HLMWs, mean loss rate: CT26, 27.1%; LLC, 26.2%), gastrocnemius weights (mean loss rate: CT26, 33.1%; LLC, 27.4%), and soleus weights (mean loss rate: CT26, 26%; LLC, 21%) as well as muscle strengths compared to the NOR mice (Fig. 1d–h; Fig. 2d–h). Compared to the NOR mice, the CAC mice exhibited obvious myasthenia with the dramatic decline (CT26, 29.6%; LLC, 27.1%) in grip strength (Fig. 1 h; Fig. 2 h). In both the CT26 model and the LLC model, AM mice exhibited alleviated cachexia features as reflected by maintained body weights, TFBWs, HLMWs, and gastrocnemius weights as well as improved muscle strengths compared with the CAC mice. Besides, the amiloride treatment significantly increased soleus weights in the CT26 cachexia model, but not observably changed those in the LLC cachexia model (Fig. 1; Fig. 2). The cross-section area analysis revealed a decreased myofiber size in gastrocnemius of the CAC mice (cachectic gastrocnemius), indicating the muscle dysfunction condition in the CT26 and CT26 cachexia models (Fig. 3a–d). As is known, skeletal muscle mass is influenced by the counter-balance between muscle degradation and myogenesis. In cachectic gastrocnemius, the decreased ratio of p-FoxO3a/FoxO3a (Fig. S2) distinctly upregulated the expressions of Atrogin-1 and MuRF-1 (Fig. 3e, f). The amiloride treatment significantly downregulated the levels of Atrogin-1 and MuRF-1 in gastrocnemius (Fig. 3e, f). Interestingly, expressions of MyoD1 and Myogenin were not statistically significantly changed both of which regulated muscle cell growth and differentiation (Fig. 3e, f). In general, the amiloride treatment preserved muscle weights and muscle strengths in tumor-bearing mice.

### Amiloride treatment alleviated cachexia-related features in mice

**Fig. 4:**
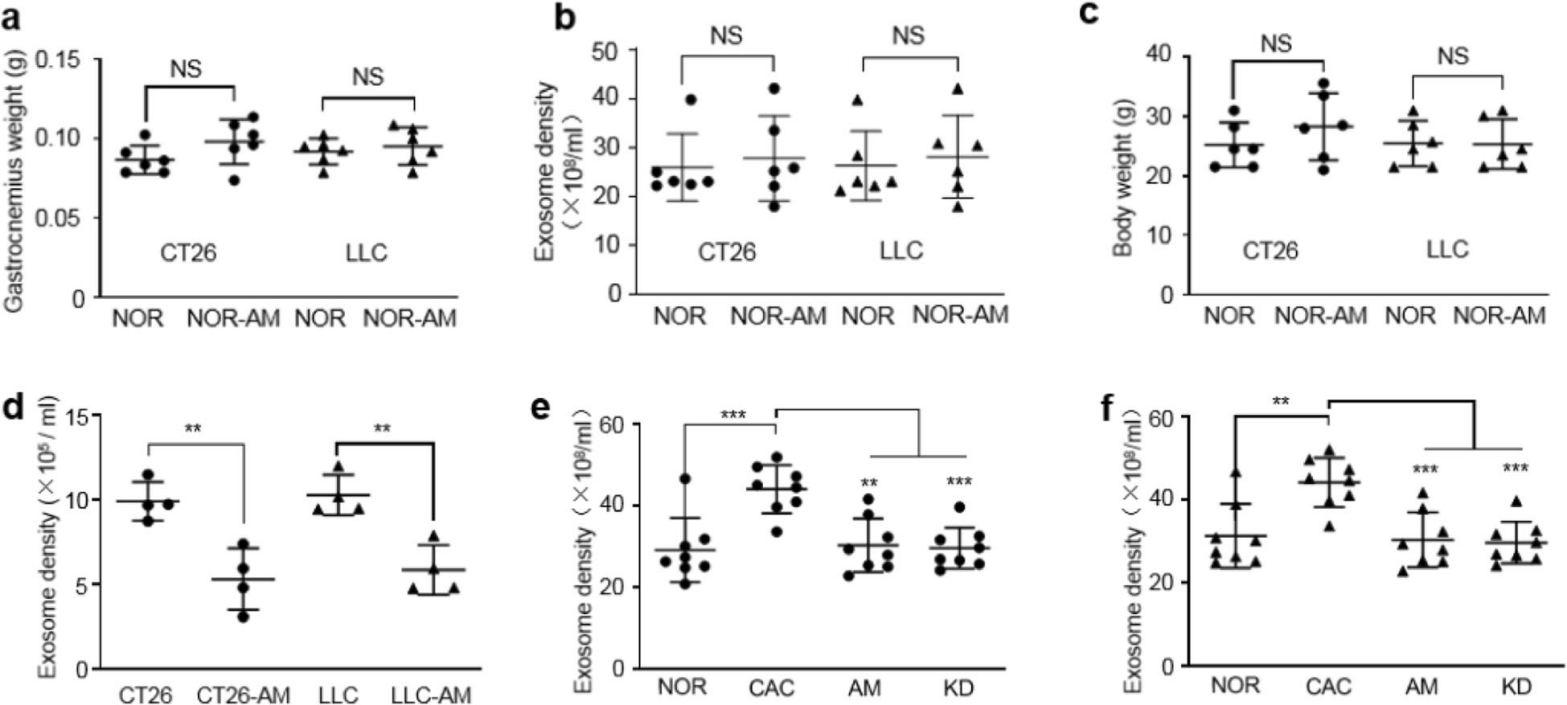
Amiloride inhibited tumor-derived exosome release both in vitro and in vivo. **a** Gastrocnemius weights of the NOR and NOR-AM mice in the CT26/LLC models (n = 6). **b** Plasma exosome densities of the NOR and NOR-AM mice in the CT26/LLC models (n = 6). **c** Body weights of the NOR and NOR-AM mice in the CT26/LLC models (n = 6). **d** Exosome densities in culture media of the CT26, CT26-AM, LLC, and LLC-AM cells (n = 4). Cells were treated by amiloride at 10 μM for 6 h. **e** Plasma exosome densities of the NOR, CAC, AM, and KD mice in the CT26 model (n = 8). **f** Plasma exosome densities of the NOR, CAC, AM, and KD mice in the LLC model (n = 8). Statistical significances: *p* > 0.05, NS; *p* < 0.05, *; *p* < 0.01, **; *p* < 0.001, ***. NOR, C57BL/6 or BALB/c normal control mice; NOR-AM, amiloride-treated NOR mice; CAC, CT26/LLC cachexia mice; AM, amiloride-treated CT26/LLC mice; KD, mice inoculated with *Rab27*-knockdown CT26/LLC cells; CT26-AM, amiloride-treated CT26 cells; LLC-AM, amiloride-treated LLC cells

Cachexia is usually accompanied by fat loss. We analyzed epididymal adipose tissues in the CT26 model and the LLC model, especially white adipose tissues which are responsible for the organismal energy storage. Compared with the NOR mice, the CAC mice displayed observable loss of epididymal adipose tissues, which was partially restored in the AM mice (Fig. 3a, b; Fig. 4a, b).

On the other hand, cachexia is mostly associated with systemic inflammatory response. In both the CT26 model and the LLC model, the CAC mice showed a profoundly upregulated serum level of IL-6 and almost identical serum levels of TNF-α and IL-1β compared with the NOR mice (Fig. S3c-e; Fig. S4c-e). Remarkably, the AM mice displayed declined serum level of IL-6 but nearly unchanged serum levels of TNF-α and IL-1β compared to the CAC mice in both models (Fig. S3c-e; Fig. S4c-e). Furthermore, the expression of down-stream phosphorylated Stat3 was remarkably upregulated in gastrocnemius of the CAC mice (CAC gastrocnemius), corresponding to the profoundly upregulated serum IL-6 level. Significantly, the expression of phosphorylated Stat3 was downregulated in gastrocnemius of the AM mice (AM gastrocnemius; Fig. S2). In addition, given that the p38 kinase participates in the cellular response to multiple stresses and inflammatory cytokines, we analyzed expressions of phosphorylated p38 and cleaved caspase-3, and observed significant enhancements of p38 kinase and caspase-3 activities in the CAC gastrocnemius, which was partially reversed in the AM gastrocnemius (Fig. S2).

Considering that anorexia is a crucial factor of cachexia, we continuously monitored food intakes across the four groups of mice. In the CT26 model, the CAC mice showed a slight decrease in average food intake compared with the other three groups of mice (Fig. S3f). Differently, in the LLC model, the CAC mice showed an average food intake similar to the other three groups of mice (Fig. S4f). Besides, both CT26 and LLC models did not exhibit observable differences in heart weight among the four groups of mice (Fig. S3g; Fig. S4g).

### Amiloride treatment ameliorated muscle wasting through inhibiting tumor-derived exosome release

We further investigated molecular mechanisms underlying the favorable anti-cachexia effects of the amiloride treatment. The AM mice exhibited similar tumor weights to the CAC mice in both the CT26 model and LLC model (Fig. 1c; Fig. 2c). Furthermore, the weight of the NOR-AM gastrocnemius did not show statistically significant change relative to the NOR gastrocnemius in both models (Fig. 4a).

Given that amiloride can exert inhibitory effects on the cellular exosome release [12, 13], we treated the CT26 and LLC cells with amiloride at various concentrations (1–200 μM) for 6 h (plasma half-life of amiloride is about 6–9 h) and did not observe statistically significant changes in the viabilities of both tumor cells (Fig. S5). We isolated exosomes from culture media of the CT26 cells, CT26-AM cells, LLC cells, and LLC-AM cells and characterized the four groups of tumor-derived exosomes (CT26 exosomes, CT26-AM exosomes, LLC exosomes, LLC-AM exosomes). Significantly, the treatment of amiloride at 10 μM for 6 h evidently inhibited exosome release from the CT26 and LLC tumor cells (Fig. 4d), indicating that the amiloride treatment profoundly decreased exosomes produced by the tumor cells.

Furthermore, we isolated exosomes from plasma of the four groups of mice in the CT26 model and the LLC model and quantified plasma exosome densities. Notably, the CAC mice showed dramatically increased plasma exosome densities compared to the NOR mice in both models (Fig. 4e, f). More importantly, the AM mice exhibited distinctly reduced plasma exosome densities in the two cachexia models (Fig. 4e, f), indicating that the amiloride treatment efficiently inhibited tumor-derived exosome release. Note that the amiloride treatment did not significantly influence the normal tissue-derived exosome release as indicated by the statistical comparison of plasma exosome density between the NOR-AM mice and the NOR mice (Fig. 4b). Besides, the NOR-AM mice did not show observable muscular toxicities as reflected by basic unchanged body weights and gastrocnemius weights compared to the NOR mice (Fig. 4a, c).

We isolated and characterized exosomes from plasma of the CAC patients and the Non-CAC patients (CAC exosomes, non-CAC exosome; Fig. S6-8). Based on the limited patient samples used in this study, we found that exosome densities in patient plasma varied even by 20-fold among individuals (Fig. S8b). In addition, the CAC patients did not display significant statistical differences in plasma exosome density from the Non-CAC patients (Fig. S8b).

On the other hand, the exosomes derived from both the CT26/LLC culture media and the CAC patient plasma induced apparent myotube atrophy in vitro*,* as indicated by the obviously decreased myotube diameters (Fig. S8a-d, h-i) and distinctly enhanced expressions of Atrogin-1 and MuRF-1 relative to PBS controls (Fig. S9). Our observation that plasma exosomes of the CAC patients profoundly promoted myotube atrophy, supporting the previous studies [7, 21].

To further confirm the experimental observation that tumor-derived exosomes significantly induced muscle atrophy in cancer cachexia, we constructed *Rab27* knock-down CT26 and LLC cell lines (CT26-KD cells, LLC-KD cells) as positive controls (Fig. S8e, f). Expectedly, knock-down of *Rab27a* and *Rab27b* potently inhibited exosome release from tumor cells without remarkably changing viabilities of the tumor cells (Fig. S8g; Fig. S10). The exosomes isolated from culture media of the CT26-KD and LLC-KD tumor cells displayed markedly reduced abilities to induce myotube atrophy relative to their corresponding wild-type counterparts (Fig. S8h, i; Fig. S9b). As expected, the KD mice inoculated with the *Rab27* knock-down tumor cells displayed alleviated cachexia features, as indicated by increased body weights, TFBWs, HLMWs, and gastrocnemius weights as well as improved muscle strengths compared with the CAC mice in the two models (Fig. 1; Fig. 2). Furthermore, the CAC, AM, and KD mice of both models showed similar tumor weights on day 30 (Fig. 1c; Fig. 2c), indicating that amiloride inhibited tumor-derived exosome release without significantly affecting tumor growth in vivo. These results indicated that the amiloride treatment could profoundly ameliorate cachectic gastrocnemius atrophy and thereby alleviate cancer cachexia through inhibiting tumor-derived exosome release.

### Amiloride treatment improved hyper-catabolism in gastrocnemius

To mechanistically understand metabolomic features of skeletal muscles in the four groups of mice, we conducted NMR-based metabolic profiling of gastrocnemius. A total of 32 metabolites were identified (Fig. S11a; Table S2). The resonance assignments were confirmed by using 2D ^1^H-^13^C HSQC spectra (Fig. S11b).

**Fig. 5:**
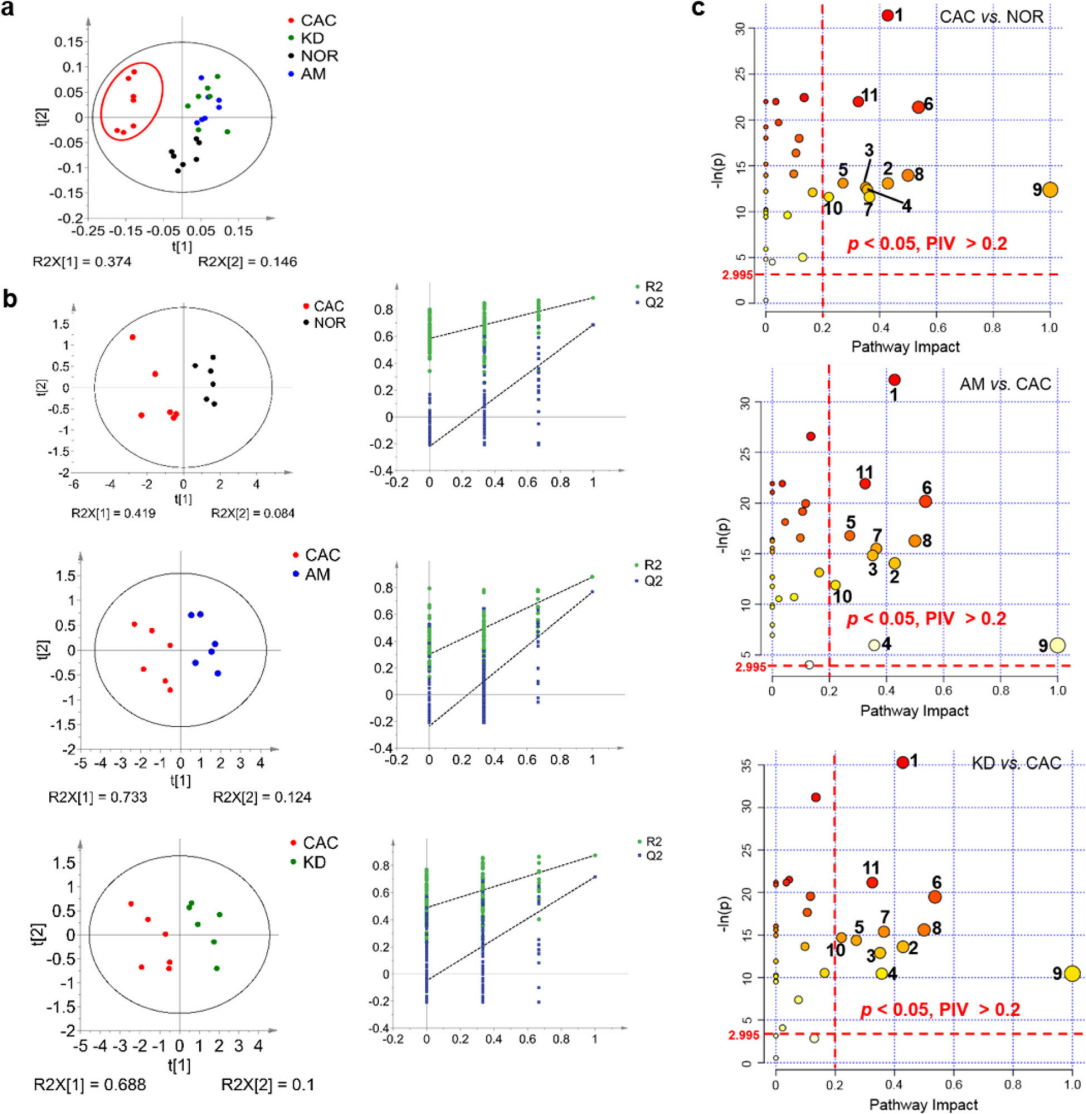
Metabolomic analyses of the NOR, CAC, AM and KD gastrocnemius. **a** PCA scores plot of 1D ^1^H NMR data obtained on aqueous extracts derived from gastrocnemius of the NOR, CAC, AM and KD mice (*n* = 7). **b** PLS-DA scores plots and cross-validation plots of 1D ^1^H-NMR data obtained from gastrocnemius of the NOR, CAC, AM and KD mice. Top: CAC mice vs. NOR mice; Middle: AM mice vs. CAC mice; Bottom: KD mice vs. CAC mice. The PLS-DA models were cross-validated to evaluate their robustness with random permutation tests (200 cycles). **c** Metabolic pathway analyses of CAC vs. NOR, AM vs. CAC, KD vs. CAC, using the Pathway Analysis module provided by MetaboAnalyst 4.0. Numbers in the three panels represent significantly altered metabolic pathways, which were identified with -ln(*p*) > 2.995 (corresponding to *p* < 0.05) and pathway impact value > 0.2: 1, taurine and hypotaurine metabolism; 2, nicotinate and nicotinamide metabolism; 3, pyruvate metabolism; 4, phenylalanine metabolism; 5, glycine, serine and threonine metabolism; 6, alanine, aspartate and glutamate metabolism; 7, glutathione metabolism; 8, D-glutamine and D-glutamate metabolism; 9, phenylalanine, tyrosine and tryptophan biosynthesis; 10, histidine metabolism; 11, starch and sucrose metabolism. NOR, C57BL/6 or BALB/c normal control mice; CAC, CT26/LLC cachexia mice; AM, amiloride-treated mice; KD, mice inoculated with Rab27-knockdown CT26/LLC cells

The unsupervised principal component analysis (PCA) scores plot illustrates that the metabolic profile of the CAC gastrocnemius is distinctly different from those of the NOR, AM and KD gastrocnemius (Fig. 5a). Similarly, the supervised partial least-squares discriminant analysis (PLS-DA) scores plots display significant metabolic distinctions in gastrocnemius between the NOR and CAC mice, the CAC and AM mice, and the CAC and KD mice (Fig. 5b). Significant metabolites were identified from the PLS-DA models (Fig. S12). Furthermore, we performed univariate analysis to quantitatively compare metabolite levels among the four groups of gastrocnemius and identify differential metabolites (Table S3).

Combining the significant metabolites with the differential metabolites, we determined characteristic metabolites for the four groups of mouse gastrocnemius (Table S4). Totally, 18, 20, and 22 characteristic metabolites were identified in gastrocnemius for CAC vs. NOR, AM vs. CAC, and KD vs. CAC, respectively. Among 17 shared characteristic metabolites, 10 metabolites (glucose, IMP, glycine, creatine, methylmalonate, niacinamide, aspartate, glutamate, fumarate and tyrosine) were increased in the CAC gastrocnemius but consistently decreased in the AM and KD gastrocnemius, and 7 metabolites (lactate, taurine, inosine, alanine, 2-phosphoglycerate, ATP and glutamine) were decreased in the CAC gastrocnemius but consistently increased in the AM and KD gastrocnemius (Table S4).

**Fig. 6:**
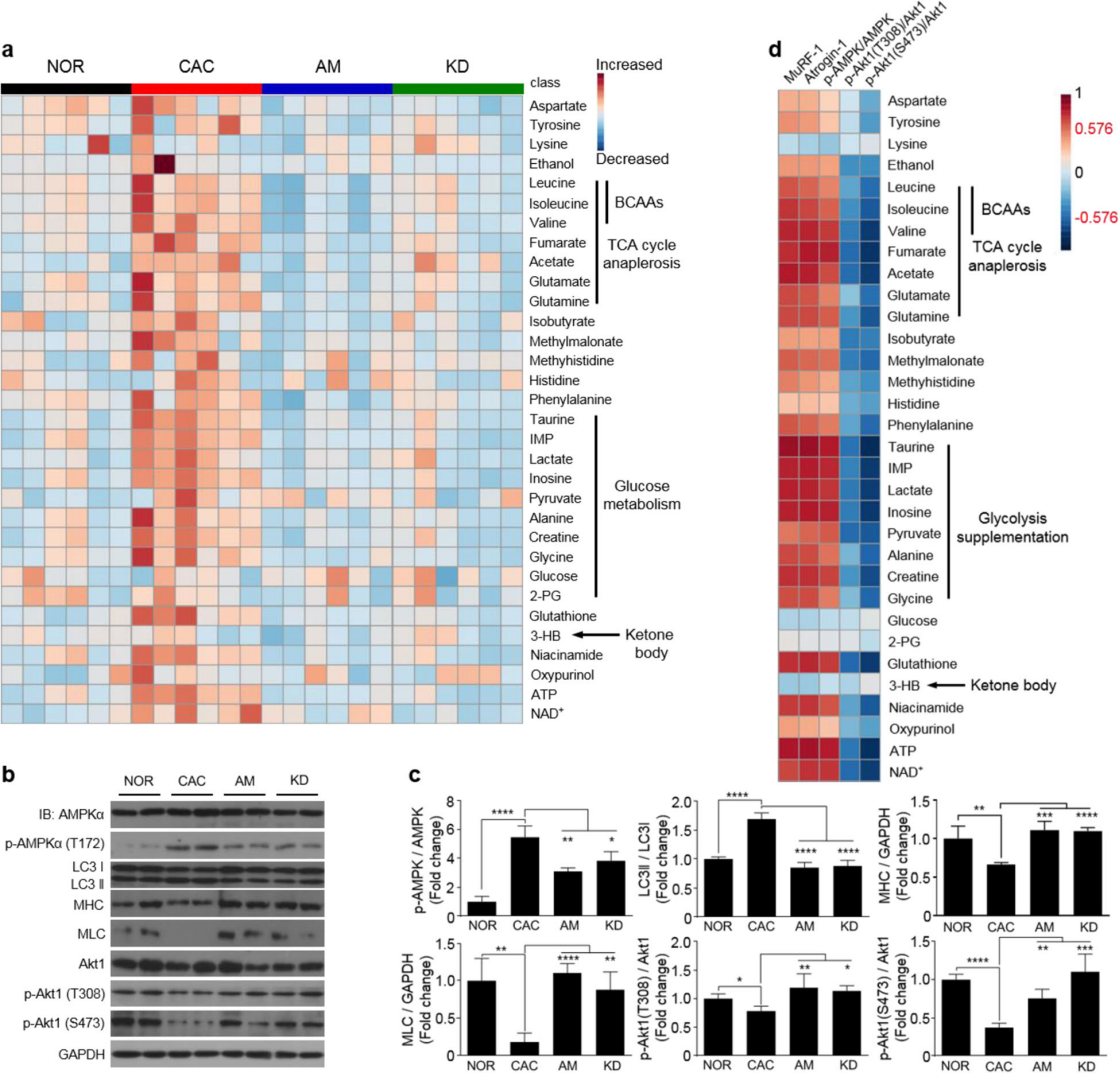
Metabolomic analyses showed that the amiloride treatment attenuated hyper-catabolism in cachectic gastrocnemius. **a** Heat-map plot of relative levels of the identified metabolites (n = 6). **b** Expressions of AMPK, p-AMPK, LC3, MHC, MLC, Akt1, p-Akt1(T308), and p-Akt1(S473) proteins analyzed by using western blot. **c** Quantification of the expressed proteins (*n* = 4). **d** Heat-map plot of the correlations between the catabolic/anabolic protein expressions and identified metabolite levels in the CAC gastrocnemius. The gradient red/blue colors indicate that the positive/negative correlations, and significant correlations were identified with the criterion of |r| > 0.576 (*n* = 6). Statistical significances: *p* > 0.05, NS; *p* < 0.05, *; *p* < 0.01, **; *p* < 0.001, ***; *p* < 0.0001, ****. NOR, C57BL/6 or BALB/c normal control mice; CAC, CT26/LLC cachexia mice; AM, amiloride-treated mice; KD, mice inoculated with Rab27-knockdown CT26/LLC cells; BCAAs, branch-chain amino acids; IMP, inosine monophosphate; 2PG, 2-phosphoglycerate; 3-HB, 3-hydroxybutyrate; MLC, myosin light chain; MHC, myosin heavy chain

Furthermore, significant pathways were identified by performing the metabolic pathway analysis for the four groups of gastrocnemius (Fig. 5c). Notably, both the amiloride treatment and *Rab27* knock-down significantly altered the identical metabolic pathways. Interestingly, 8 of the 11 significant pathways were closely related to amino acid metabolism. The heat-map plot of relative metabolite levels illustrates that a large number of amino acids were increased in the CAC gastrocnemius, including alanine, glutamine, aspartate, and glycine (Fig. 6a). The upregulated levels of phosphorylated AMPK-Tyr112 and downregulated levels of phosphorylated Akt1 indicated a strongly activated catabolism in cachectic gastrocnemius (Fig. 6b, c). Moreover, the raised ratio of LC3II/LC3I and declined levels of MHC and MLC were indicative of promoted autophagy in cachectic gastrocnemius (Fig. 6b, c). Additionally, the levels of upregulated amino acids were positively correlated with expressions of catabolic proteins, but negatively correlated with expressions of anabolic proteins in the CAC gastrocnemius (Fig. 6d).

**Fig. 7:**
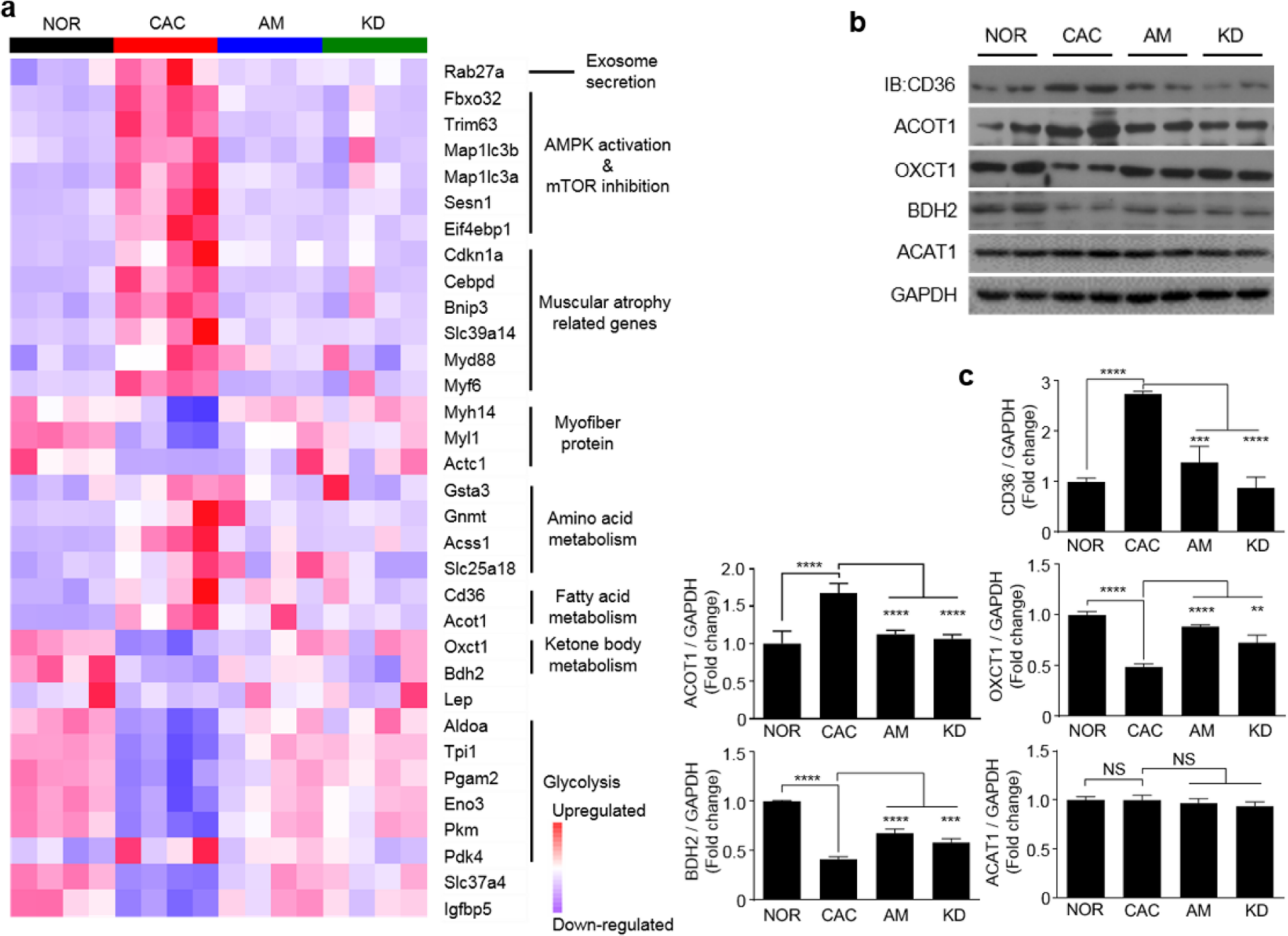
Transcriptomic analyses exhibited that the amiloride treatment improved glycolysis and ketone body oxidation in cachetic gastrocnemius. **a** Heat-map plot of relative transcription levels of muscular atrophy-related genes (n = 4). **b** Expressions of ACOT1, CD36, OXCT1, BDH2, and ACAT1 proteins. **c** Quantification of the expressed proteins (n = 4). Statistical significances: *p* > 0.05, NS; *p* < 0.05, *; *p* < 0.01, **; *p* < 0.001, ***; *p* < 0.0001, ****. NOR, C57BL/6 or BALB/c normal control mice; CAC, CT26/LLC cachexia mice; AM, amiloride-treated mice; KD, mice inoculated with *Rab27*-knockdown CT26/LLC cells; ACOT1, acyl-coenzyme A thioesterase 1; CD36, fatty acid translocase; OXCT1, 3-oxoacid CoA transferase 1; BDH2, 3-hydroxybutyrate dehydrogenase 2; ACAT1, acetyl coenzyme A acetyltransferase 1

Transcriptomic profiling identified 1466 upregulated genes and 1941 downregulated genes in the CAC gastrocnemius relative to the NOR gastrocnemius, of which 753 differentially expressed genes (DEGs) were shared by the pairwise comparisons of CAC vs. NOR, AM vs. CAC, and KD vs. CAC (Fig. S13a; Fig. S14a). The KEGG enrichment analysis based on the identified DEGs screened out distinctly changed metabolism-related processes including carbohydrate metabolism, lipid metabolism, and amino acid metabolism (Fig. S13b; Fig. S14b). Consistently, the loss of epididymal adipose tissues in cachexia mice (Fig. S2a, b; Fig. S3a, b) was accompanied by upregulated expressions of fatty acid translocase *CD36* and Acyl-coenzyme A thioesterase 1 (*Acot1*) in the CAC gastrocnemius (Fig. 7b, c; Table S5), which facilitated mobilization and oxidation of adipose tissues.

Overall, the amiloride treatment substantially improved hyper-catabolism in cachectic gastrocnemius, and *Rab27* knock-down showed a similar improvement of hyper-catabolism (Fig. 6; Fig. 7).

### Amiloride treatment improved blocked glycolysis and impeded ketone body oxidation in gastrocnemius

Glycolysis is one of the fundamental energy sources in muscle cells, and ketone bodies are alternative energy sources under harsh conditions. Glucose was significantly increased in the CAC gastrocnemius relative to the NOR gastrocnemius, contrarily, the glycolysis end product—pyruvate—was decreased (Table S3). The expressions of multiple glycolytic catalyzing enzymes were downregulated, but the expression of glycolytic inhibition enzyme (pyruvate dehydrogenase kinase isoenzyme 4, *Pdk4*) was upregulated in cachectic gastrocnemius relative to normal control (Fig. 7a; Table S5). Furthermore, the CAC gastrocnemius exhibited more than 2-fold decreases in expressions of ketone body oxidation enzymes 3-oxoacid CoA transferase 1 (OXCT1) and 3-hydroxybutyrate dehydrogenase 2 (BDH2; Fig. 7b,c) but substantially unchanged expressions of ketone body transporters (MCT1 and MCT4; Fig. S2) and the dominant organismal ketone body—3-hydroxybutyrate (3-HB; Table S3). These results were indicative of blocked glycolysis and impeded ketone body oxidation in the CAC gastrocnemius. Significantly, both the AM and KD gastrocnemius displayed profoundly improved glycolysis and ketone body oxidation via the inhibition of tumor-derived exosome release (Fig. 7; Fig. S15).

## Discussion

**Fig. 8:**
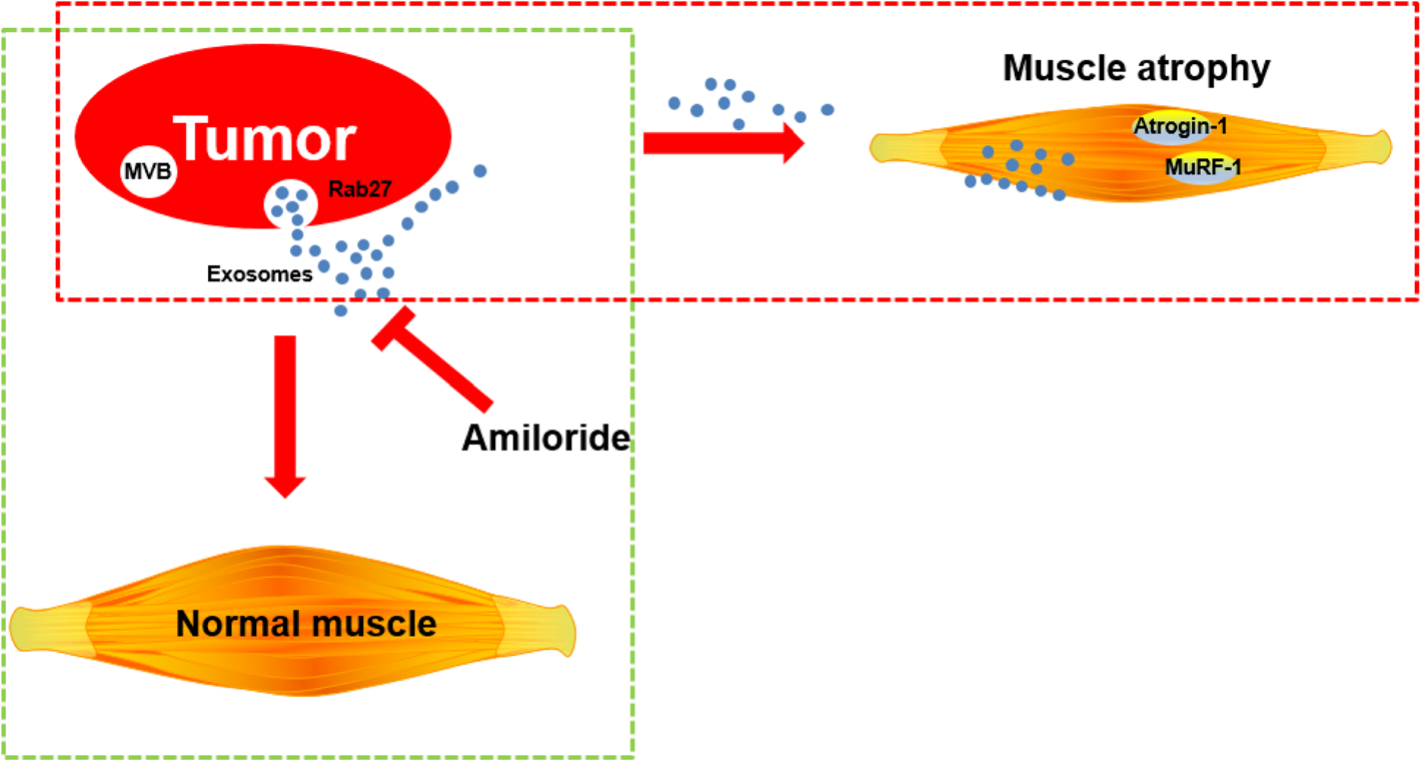
Graphic model of the amiloride treatment ameliorating cachectic muscle wasting through inhibiting tumor-derived exosome release

Cancer cachexia evidently reduces patient survival and quality of life due to its high incidence and mortality rate [4, 22]. Developments of efficient therapeutic strategies are urgently required for the CAC treatments. Previous studies have demonstrated that amiloride possesses potassium-sparing diuretic function, which has been clinically used in the treatments of hypertension and edema due to heart failure [11, 23–25]. Moreover, amiloride can inhibit exosome release from cells and reverse exosome-promoted pathogenic processes [26, 27]. In this study, we established CT26/LLC-induced mouse models of lung/colorectal cancer cachexia, assessed the effects of the amiloride treatment for alleviating muscle atrophy in the two cachexia models, and addressed the underlying molecular mechanisms. Our results reveal that amiloride is a potential therapeutic drug capable of ameliorating muscle wasting in cancer cachexia through inhibiting tumor-derived exosome release (Fig. 8).

Both the CT26 model and the LLC models showed remarkable cachexia features and significant metabolic impairments in gastrocnemius. Significantly, the amiloride treatment prevented the losses of body weight, skeletal muscle, and fat mass, which did not obviously affect tumor growth and induce observable systemic toxicities in normal control mice, as indicated by basically unchanged body weights and gastrocnemius weights of the NOR-AM mice relative to the NOR mice. More importantly, multiple cachexia features were improved, as evidenced by downregulated expressions of muscular atrophic proteins, partially restored muscle strength and neutralized systemic inflammation. The further mechanistic study revealed that the amiloride treatment profoundly inhibited tumor-derived exosome release and attenuated hyper-catabolism, significantly improved the metabolic impairments in cachectic gastrocnemius, thereby alleviating the CAC progression.

By integrative metabolomic and transcriptomic analyses, we identified significantly impaired metabolic pathways in cachectic gastrocnemius relative to normal control, including promoted muscular catabolism, inhibited muscular protein synthesis, blocked glycolysis, and impeded ketone body oxidation. Expectedly, the impaired metabolic pathways potentially contribute to metabolic mechanisms underlying the CAC progression. Previously, we established a mouse model of gastric cancer cachexia by orthotopically implanting BGC823 cells and identified significantly impaired metabolic pathways in cachectic gastrocnemius [19]. The two works of ours unanimously confirm that aberrant catabolism in cachectic gastrocnemius is triggered primarily by upregulated E3 ligases, which exhibit profoundly increased levels of amino acids (isoleucine, leucine, valine, glutamate). Interestingly, the CT26/LLC cachexia models show blocked glycolysis in gastrocnemius, while the BGC823 cachexia model displays promoted glycolysis. The metabolic distinction might be dictated by several factors including differences in food intake, inflammatory cytokines, tumor type, and stage of cachexia. Additionally, the discrepancy between orthotopic and subcutaneous cachexia models might be associated with discrepant metabolic features in gastrocnemius, potentially contributing to the metabolic distinction, even though these models undergo similar muscular atrophic processes.

In the past decades, strategies for cachexia therapy mainly focused on the development of new drugs, including ghrelin and ghrelin receptor agonists, myostatin antagonists, inflammatory cytokine neutralizing antibodies, and natural product extracts [28–37]. Furthermore, recent studies have identified several key molecules (namely cachectin) and correspondingly explored inhibitory chemicals for alleviating the CAC progression. However, most of these efforts have not obtained satisfactory clinical trial results [38]. The primary reason might attribute to the heterogeneity of cachectin resulting from either different tumor types or being aroused by inherent reprogramming processes within identical tumor types, which requires more comprehensive investigation. Besides, it is time-consuming for clinical trials and further approval of newly developed drugs. In contrast, applying existing drugs for new indications could be a more feasible alternative strategy. Given that most of the existing drugs are usually associated with well addressed pharmacokinetic and pharmacodynamic properties and toxicity profiles, the alterative strategy might greatly reduce the time required to develop novel drugs for the CAC treatment.

Amiloride has been clinically used for nearly three decades in the treatments of hypertension, edema and congestive heart failure [23–25, 39]. In the present study, we demonstrated for the first time the therapeutic potentials of amiloride in the treatments of cancer cachexia. Previously, Cameron et al. showed that increased Na^+^ contents in multiple tissues of H6 hepatoma-induced cachexia mice can be partially reversed by the amiloride treatment [40]. Their study mainly determined amiloride-induced changes of Na^+^ and other ions contents in liver cachexia mice, but did not further examine whether cachexia symptoms were relieved.

The most outstanding characteristic of amiloride is the efficient inhibitory effects on the Na^+^/H^+^ and Na^+^/K^+^ transporters [11, 23, 26, 41]. Under normal conditions, the Na^+^/H^+^ transporter mediates H^+^ efflux from the cells in exchange for Na^+^ influx. Further works indicated that blocking Na^+^ influx/ H^+^ efflux can inhibit cell growth, and tumor cells are more vulnerable to the blocking of H^+^ efflux than normal cells which might endow amiloride antineoplastic effects [26, 27, 41–44].

In this study, we observed that the treatment of amiloride at various concentrations (1–200 μM) for 6 h did not observably change cell viabilities of tumor cells Moreover, both the CT26 model and the LLC model showed that the amiloride treatment did not statistically significantly affect tumor growth in the CAC mice. These results suggest that the antineoplastic effects of amiloride do not significantly contribute to amiloride-mediated alleviation of the CAC progression.

On the other hand, intracellular Na^+^ contents also regulate the trafficking of extracellular vesicles including exosomes [45]. The Na^+^/Ca^2+^ antiporter mediates the appropriate Na^+^ efflux in exchange for Ca^2+^ influx. The increased cytoplasmic Ca^2+^ content is a prerequisite for multi-vesicular bodies (MVBs) generation and following exosome biogenesis [40, 45]. Thus, amiloride probably decreases intracellular Ca^2+^ content through mediating Na^+^ efflux by the antiporter, and obstructs cellular exosome release. Note that we could not currently confirm this speculation as this study had not measured the contents of Ca^2+^ and Na^+^.

Both the CL26 and LLC murine models showed dramatic decreases in plasma exosome densities of the AM mice relative to the CAC mice. Note that plasma exosomes could also be derived from normal tissues besides tumor tissues. The amiloride treatment (2 mg/kg/day) did not statistically change plasma exosome densities derived from normal tissues in the normal control mice, as indicated by the observation that the NOR-AM mice and the NOR mice did not show statistically significant difference in plasma exosome density. Thus, it could be expected that the decreases in plasma exosome density in the amiloride-treated CAC mice might reflect the reductions in exosomes produced by the tumor cells. Furthermore, the quantitative analysis of exosome densities in culture media of the CT26/LLC tumor cells indicated that the amiloride treatment (10 μM, 6 h) profoundly deceased exosome release from the tumor cells but not observably affected viabilities of the tumor cells. These experimental results reveal that the amiloride treatment significantly reduces exosome production by the tumor cells. Nevertheless, more experiments should be performed to further support the conclusion that amiloride ameliorates muscle wasting through inhibiting tumor-derived exosome release and thereby alleviates cancer cachexia. Potentially, the alleviation of cachexia could be due to several factors including reduced circulating IL-6, maintenance of cardiac function, and direct effects on skeletal muscle, etc. These factors are worthy of further exploration in future studies.

A previous study exhibited that the amiloride treatment at two doses of 10 mg/kg and 15 mg/kg did not exhibit significant toxic effects in a multiple myeloma xenograft murine model, as indicated by basically unchanged body weights [27]. Consistently, the amiloride treatment at 2 mg/kg used in our study did not show observable muscular toxic effects, as indicated by basically unchanged body weights and gastrocnemius weights in the NOR-AM mice relative to the NOR mice. However, our limited results only reflect that amiloride has not significant muscle toxicity in healthy mice. A systemic toxicity test should be conducted in further studies. Nevertheless, the amiloride treatment may be beneficial to the amelioration of cachectic muscle wasting and thus to the alleviation of the CAC progression.

Previous studies documented that cancer cachexia can be induced by multiple factors [35, 38, 46, 47], including cytokines, hormones, tumor factors, and gut microbes. More significantly, the present study displayed that the exosomes isolated from plasma of cancer cachexia patients and culture media of the CT26/LLC tumor cells induced remarkable myotube atrophy, well confirming that tumor-derived exosomes can induce muscle wasting in cancer cachexia. Furthermore, it has been demonstrated that several individual components in tumor-derived exosomes can significantly contribute to cancer cachexia progression, such as miR-21 [14], heat shock proteins [9], and metal ions [48]*.* Expectedly, exploration of other key components in tumor-derived exosomes would be greatly beneficial to further understand the molecular mechanisms of exosome-induced muscle wasting and early diagnosis of cancer cachexia. Such studies are worthy of being conducted in the future.

The present study revealed that the amiloride treatment can significantly inhibit tumor-derived exosome release, and thereby profoundly ameliorate muscle wasting and alleviate the CAC progression, indicating clinical potentials of amiloride for treatments of the CAC patients. Similar to amiloride, some other drugs or chemical inhibitors, such as GW4869 [49–51], omeprazole [12], chlorpromazine [52], and statins [53–55], also possess the effects of inhibiting cellular exosome release, and could be exploited as potential drugs against cancer cachexia too.

Cachectic muscle atrophy primarily results from an imbalance of catabolism and anabolism [2]. Amiloride-mediated inhibition of tumor-derived exosome release significantly improves metabolic impairments in cachectic gastrocnemius. As is known, both activation of the AMPK signaling cascade and inhibition of the Akt pathway are responsible for muscle wasting in cancer cachexia. Moreover, activation of the apoptosis pathway mediated by the p38 kinase also contributes to myofibrillar protein degradation and muscle dysfunction. Cachectic gastrocnemius exhibited a more than 5-fold increase in the ratio of p-AMPK (Tyr112)/AMPK and significant decreases in the ratio of p-Akt1/Akt1, indicating the promoted catabolism and inhibited anabolism in cancer cachexia. The metabonomic analysis of the CAC gastrocnemius exhibited upregulated amino acid levels relative to normal control, further confirming the promoted degradations and inhibited syntheses of muscular proteins. The accumulated amino acids are capable of acting as supplementary sources for both TCA cycle anaplerosis and glycolysis, fulfilling increased energy demand in cancer cachexia. Furthermore, the transcriptomic analysis of the CAC gastrocnemius showed downregulated expressions of five glycolytic catalyzing enzymes and the upregulated expression of a glycolytic inhibiting enzyme relative to the NOR gastrocnemius, indicating that blocked glycolysis significantly promoted muscle wasting in cancer cachexia. More significantly, amiloride-mediated inhibition of tumor-derived exosome release enhances the promoted muscular proteolysis, inhibited muscular protein synthesis, and blocked glycolysis in cachectic gastrocnemius.

In addition, we found that tumor-derived exosomes are also involved in the regulation of ketone body metabolism in skeletal muscle. In harsh energy conditions (continuous intensive exercise training; constant hunger, etc.), ketone bodies are mostly transported across the blood-brain barrier to fuel the brain. In cachexia mice, hepatogenic ketone bodies are available energy sources for extrahepatic tissues (mainly in the brain and skeletal muscles). We detected downregulated expressions of key enzymes (BDH2 and OXCT1) for ketone body oxidation in cachectic gastrocnemius, implying that ketone body oxidation was potentially impeded. Consistently, the inhibition of tumor-derived exosome release by the amiloride treatment can alleviate the impediment of ketone body oxidation in cachectic gastrocnemius. We thus speculate that the impeded ketone body oxidation might attribute to a potential protective mechanism for ensuring the preferential supply of ketone bodies to the brain. Other alternative energy sources such as amino acids and acetyl-CoAs are available for other organs to sustain energy production and biomolecules synthesis. Here, we could not confirm this speculation as we did not assess the ketone body utilization in the brain of cachexia mice. Nevertheless, our study could be an innovative supplementation for clarifying the molecular mechanisms of skeletal muscle atrophy in cancer cachexia. Expectedly, it is of great value to exploit ketone body metabolism-related enzymes as novel targets for improving ketone body utilization and thereby ameliorating cachectic muscle wasting. It seems that enhancing ketone body utilization rather than simply serving cachectic mice with ketogenetic diets [56], might be a more efficient way to ameliorate muscle wasting in cancer cachexia.

## Conclusions

We have demonstrated that amiloride is a potential drug capable of ameliorating muscle wasting in cancer cachexia. Our results reveal that the amiloride treatment significantly improves metabolic impairments in cachectic gastrocnemius through efficiently inhibiting tumor-derived exosome release, including promoted muscular catabolism, inhibited muscular protein synthesis, blocked glycolysis, and impeded ketone body oxidation. Our results are beneficial to mechanistic understanding the effects of the amiloride treatment for ameliorating muscle wasting in cancer cachexia and alleviating the CAC progression. Our study sheds light on the potentials of amiloride in cachexia therapy. Further studies are needed both to validate the practical universalities of the amiloride treatment for other cancer cachexia models, and to explore clinical potentials of amiloride for improving the CAC treatment.

## Supplementary Information

**Additional file 1:** Supplementary Fig. S1-S15 and Tables S1-S5.

**Additional file 2:** The DEGs identified in the transcriptomics study.

**Additional file 3:** The metabolomics data obtained from the present study.

## Data Availability

Transcriptome datasets can be found with a GEO accession number: GSE173250. Other datasets supporting the conclusions of this article are included within the article and its additional files.

## Abbreviations

CAC: Cancer cachexia
NOR: Normal control
AM: Amiloride treated
KD: *Rab27* knock-down
MVBs: Multi-vesicular bodies
CM: Conditioned media
HSFCM: High-sensitivity nano flow cytometer
FID: Free induction delay
DEGs: Differentially expressed genes
FC: Fold change
FDR: False discovery rate
TFBWs: Tumor-free body weights
HLMWs: Hind limb muscle weights
PCA: Principal components analysis
PLS-DA: Supervised partial least-squares discriminant analysis
OXCT1: 3-Oxoacid CoA transferase 1
BDH2: 3-Hydroxybutyrate dehydrogenase 2
FFA: Free fatty acid
FAA: Free amino acid
KB: Ketone body
α-KG: α-Ketoglutarate
AcAc: Acetoacetic acid
F-1,6-BP: Fructose-1,6-bisphosphate
G3P: Glyceraldehyde-3-phosphate
DHAP: Dihydroxyacetone phosphate
3PG: 3-Phosphoglycerate
2PG: 2-Phosphoglycerate
PEP: Phosphoenolpyruvate
3-HB: 3-Hydroxybutyrate
IMP: Inosine monophosphate
