# Supplementary material for: Amiloride ameliorates muscle wasting in cancer cachexia through inhibiting tumor-derived exosome release
Source: Skelet Muscle. 2021 Jul 6;11:17. doi: 10.1186/s13395-021-00274-5 (PMC8258996; doi:10.1186/s13395-021-00274-5)
Supplement: Supplementary file 3 — Additional file 3: The metabolomics data obtained from the present study. [file 13395_2021_274_MOESM3_ESM.docx]

**Amiloride ameliorates muscle wasting in cancer cachexia through inhibiting tumor-derived exosome release**

Lin Zhou^1^, Tong Zhang^1^, Wei Shao^3^, Ruohan Lu^1^, Lin Wang^4^, Haisheng Liu^1^, Bin Jiang^5^, Shiqin Li^6^, Huiqin Zhuo^7^, Suheng Wang^8^, Qinxi Li^5^, Caihua Huang^2*^, Donghai Lin^1,9*^

^1^Key Laboratory for Chemical Biology of Fujian Province, MOE Key Laboratory of Spectrochemical Analysis & Instrumentation, College of Chemistry and Chemical Engineering, Xiamen University, Xiamen 361005, China,

^2^Research and Communication Center of Exercise and Health, Xiamen University of Technology, Xiamen 361024, China,

^3^Xiamen Cardiovascular Hospital, Xiamen University, Xiamen 361000, China,

^4^Department of Oncology, Institute of Gastrointestinal Oncology, Zhongshan Hospital, Xiamen University, Xiamen 361004, China,

^5^State Key Laboratory of Cellular Stress Biology, School of Life Sciences, Xiamen University, Xiamen 361102, China,

^6^Department of Medical Oncology, Xiang'an Hospital of Xiamen University, Xiamen,

^7^Department of Gastrointestinal Surgery, The Affiliated Zhongshan Hospital, Xiamen University, Xiamen, Fujian 361004, China,

^8^Collaborative Innovation Center of Chemistry for Energy Materials, College of Chemistry and Chemical Engineering, Xiamen University, Xiamen, 361005, China,

^9^High-field NMR Center, College of Chemistry and Chemical Engineering, Xiamen University, Xiamen 361005, China.

**Corresponding Authors:**

*Donghai Lin: High-field NMR Center, College of Chemistry and Chemical Engineering, Xiamen University, Xiamen 361005, China. Tel: +86-592-2186078, e-mail address: dhlin@xmu.edu.cn.

*Caihua Huang: Research and Communication Center of Exercise and Health, Xiamen University of Technology, Xiamen 361021, China. Tel: +86-592-6291679, e-mail address: huangcaihua@xmut.edu.cn

**Supplementary Material**

**
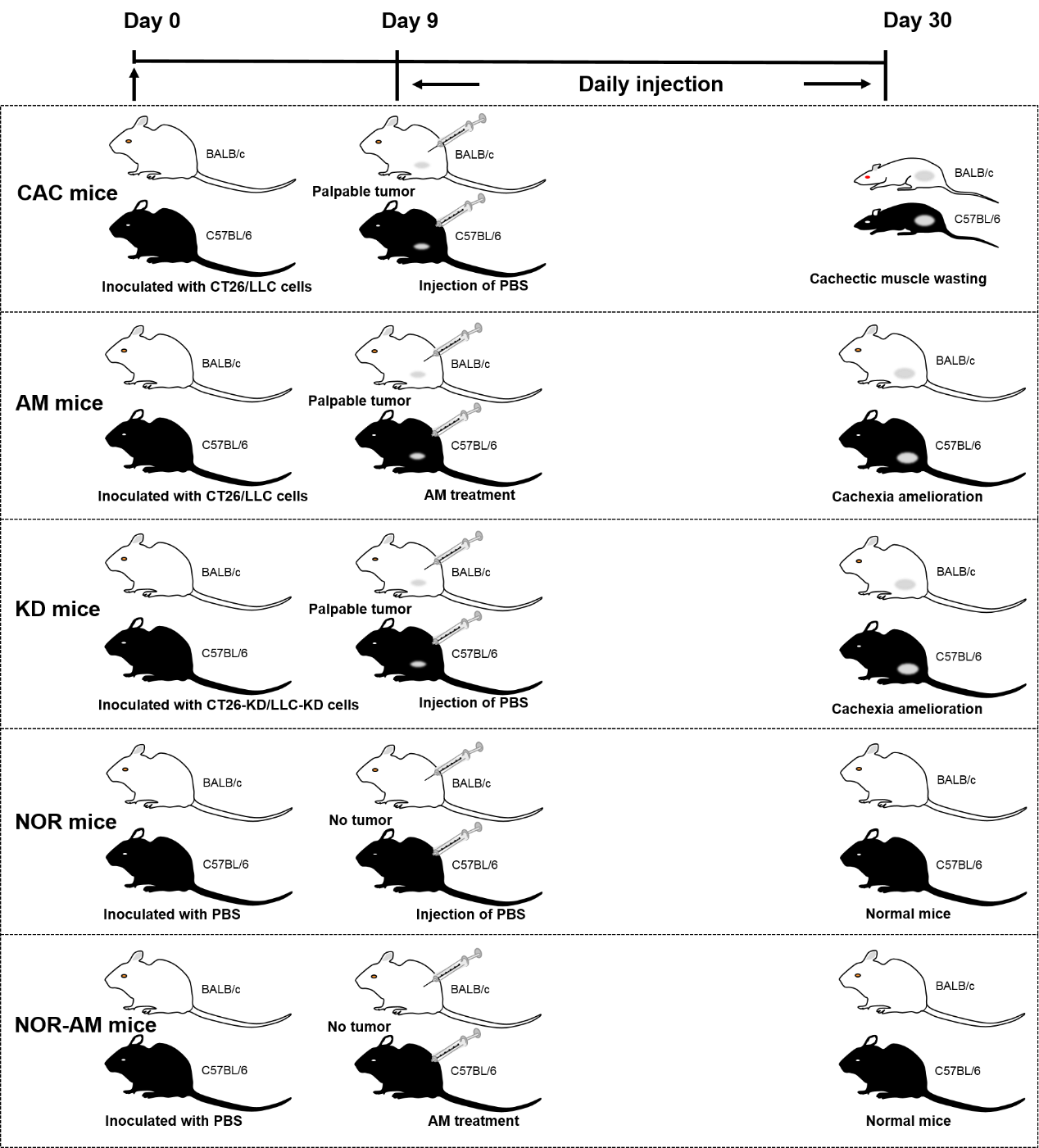
Fig. S1 Illustration of the processes of establishing murine cachexia models.** Tumor cells were subcutaneously injected into the right flank of mice on day 0. The BALB/c male and female mice were inoculated with the CT26 cells (1.0×10^6^/100 μL), while the C57BL/6 male mice were inoculated with the LLC cells (7×10^5^/100 μL). Both BALB/c and C57BL/6 mice showed palpable tumors (about 5 mm in diameter) on day 9 after the inoculation. Both the CT26-bearing mice and LLC-bearing mice were randomly divided into 2 groups (n = 8 per group): one group of mice intraperitoneally injected daily with PBS from day 9 (CAC mice), another group of mice intraperitoneally injected daily with amiloride dissolved in PBS at a dose of 2 mg/kg (AM mice). Furthermore, The *Rab27* knock-down CT26/LLC cells were subcutaneously injected into the mice following the same procedure (KD mice). Similarly, normal control mice were injected with PBS on day 0 (NOR mice). Both the KD and NOR mice were intraperitoneally injected with PBS daily from day 9. To evaluate potential toxicities of the amiloride treatment, either C57BL/6 mice or BALB/c mice were divided into 2 groups: NOR mice，NOR-AM mice, 6 per group. PBS was subcutaneously injected into the right flank of the NOR and NOR-AM mice on day 0. From day 9, the NOR-AM mice were intraperitoneally injected daily with amiloride at the same dose of 2 mg/kg following the procedure described above, whereas the NOR mice with PBS continually. On day 30, all mice were sacrificed.

**
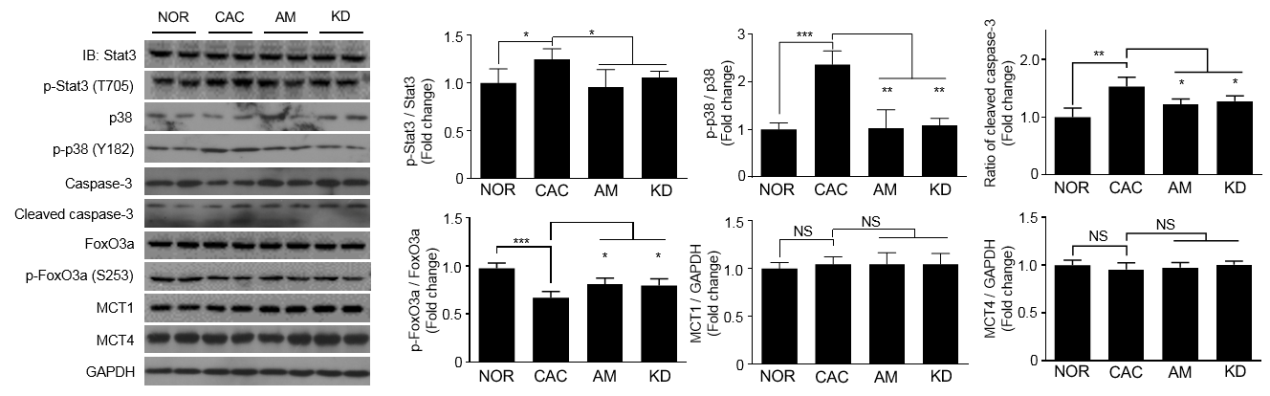
Fig. S2 Expressions of muscular atrophy-related proteins and ketone body transporter proteins in the NOR, CAC, AM and KD gastrocnemius.** Expressions of the following proteins were analyzed by using western blot (n = 4): Stat3, p-Stat3, FoxO3a, p-FoxO3a, p38, p-p38, caspase-3, cleaved caspase-3, MCT1 and MCT4. Statistically significances: *p* > 0.05, NS; *p* < 0.05, *; *p* < 0.01, **; *p* < 0.001, ***; *p* < 0.0001, ****. Abbreviations: NOR, C57BL/6 or BALB/c normal control mice; CAC, CT26/LLC cachexia mice; AM, amiloride-treated mice; KD, mice inoculated with *Rab27*-knockdown CT26/LLC cells.

**
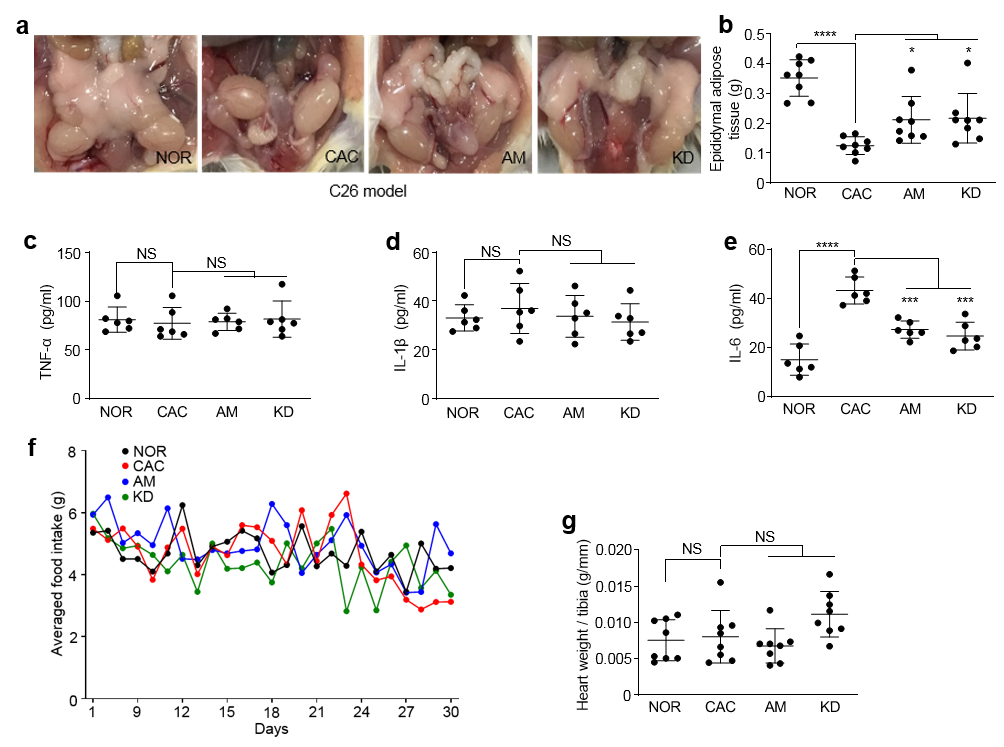
**

**Fig. S3** **Characterization of cachexia features in the NOR, CAC, AM and KD mice of the CT26 model.** (a) Representative pictures of epididymal adipose tissues of the mice. (b) epididymal adipose tissue weights (n = 8). (c-e) Serum IL-6, TNF-α and IL-1β levels of the mice (n = 6). (f) Averaged food intakes of the mice (n = 6). (g) Heart weights of the mice (n = 8). Statistically significances: *p* > 0.05, NS; *p* < 0.05, *; *p* < 0.01, **; *p* < 0.001, ***; *p* < 0.0001, ****. Abbreviations: NOR, C57BL/6 normal control mice; CAC, CT26 cachexia mice; AM, amiloride-treated mice; KD, mice inoculated with *Rab27*-knockdown CT26 cells.

**
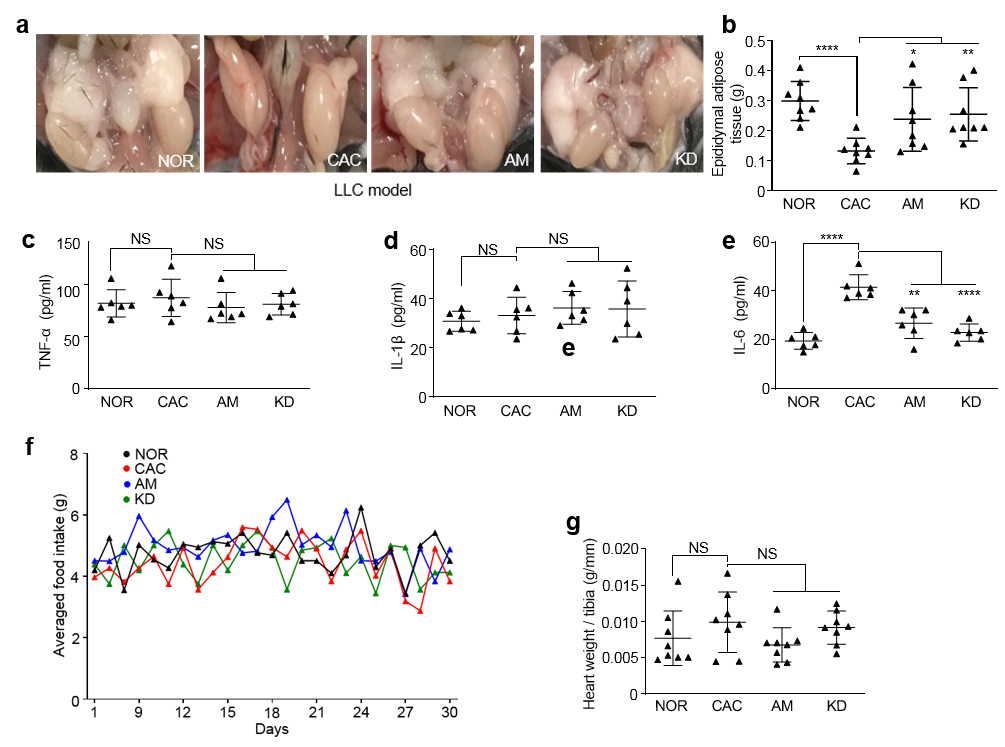
**

**Fig. S4** **Characterization of cachexia features in the NOR, CAC, AM and KD mice of the LLC model.** (a) Representative pictures of epididymal adipose tissues of the mice. (b) epididymal adipose tissue weights (n = 8). (c-e) Serum IL-6, TNF-α and IL-1β levels of the mice (n = 6). (f) Averaged food intakes of the mice (n = 6). (g) Heart weights of the mice (n = 8). Statistically significances: *p* > 0.05, NS; *p* < 0.05, *; *p* < 0.01, **; *p* < 0.001, ***; *p* < 0.0001, ****. Abbreviations: NOR, BALB/c normal control mice; CAC, LLC cachexia mice; AM, amiloride-treated mice; KD, mice inoculated with *Rab27*-knockdown LLC cells.


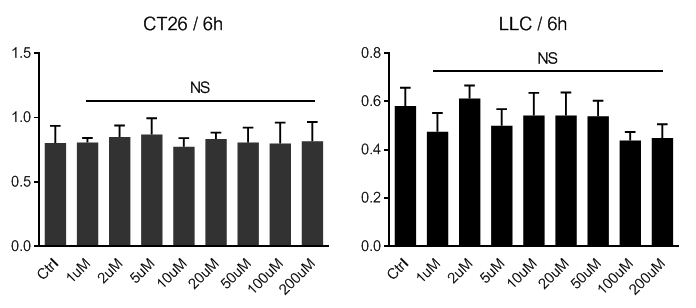


**Fig. S5** **Viabilities of amiloride-treated CT26 and LLC cells.** The CT26 and LLC cells were treated with amiloride at various concentrations (1 μM- 200 μM)) for six hours. Cell viabilities were assessed by the MTS assay (n = 4). Statistically significances: *p* > 0.05, NS; *p* < 0.05, *; *p* < 0.01, **; *p* < 0.001, ***; p < 0.0001, ****.

**
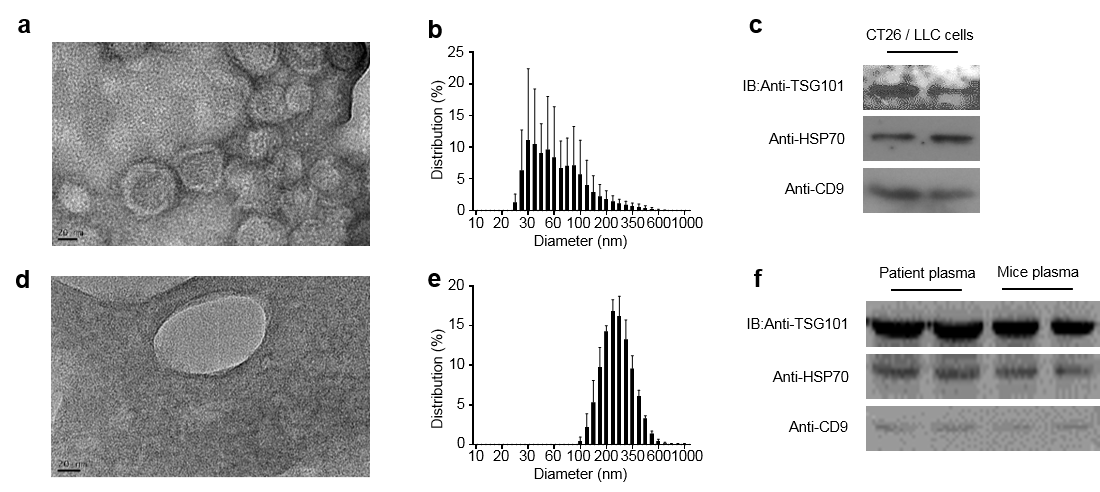
Fig. S6 Characterization of exosomes isolated both from culture media of the CT26/LLC cells and from plasma of the patients/mice.** (a) Typical transmission electron microscope pictures of the CT26/LLC exosomes (scale bar = 20 nm). (b) Diameter distributions of CT26/LLC exosomes analyzed with Zeta View® Nanoparticle Tracking Analyzer (n = 4). (c) Expressions of three exosome maker proteins (CD9, TSG101 and HSP70) analyzed by using western blot. (d) Typical transmission electron microscope picture of patient plasma exosomes (scale bar = 20 nm). (e) Diameter distribution of patient plasma exosomes analyzed with Zeta View® Nanoparticle Tracking Analyzer (n = 4). (f) Expressions of three exosome maker proteins (CD9, TSG101 and HSP70) analyzed by using western blot.

**
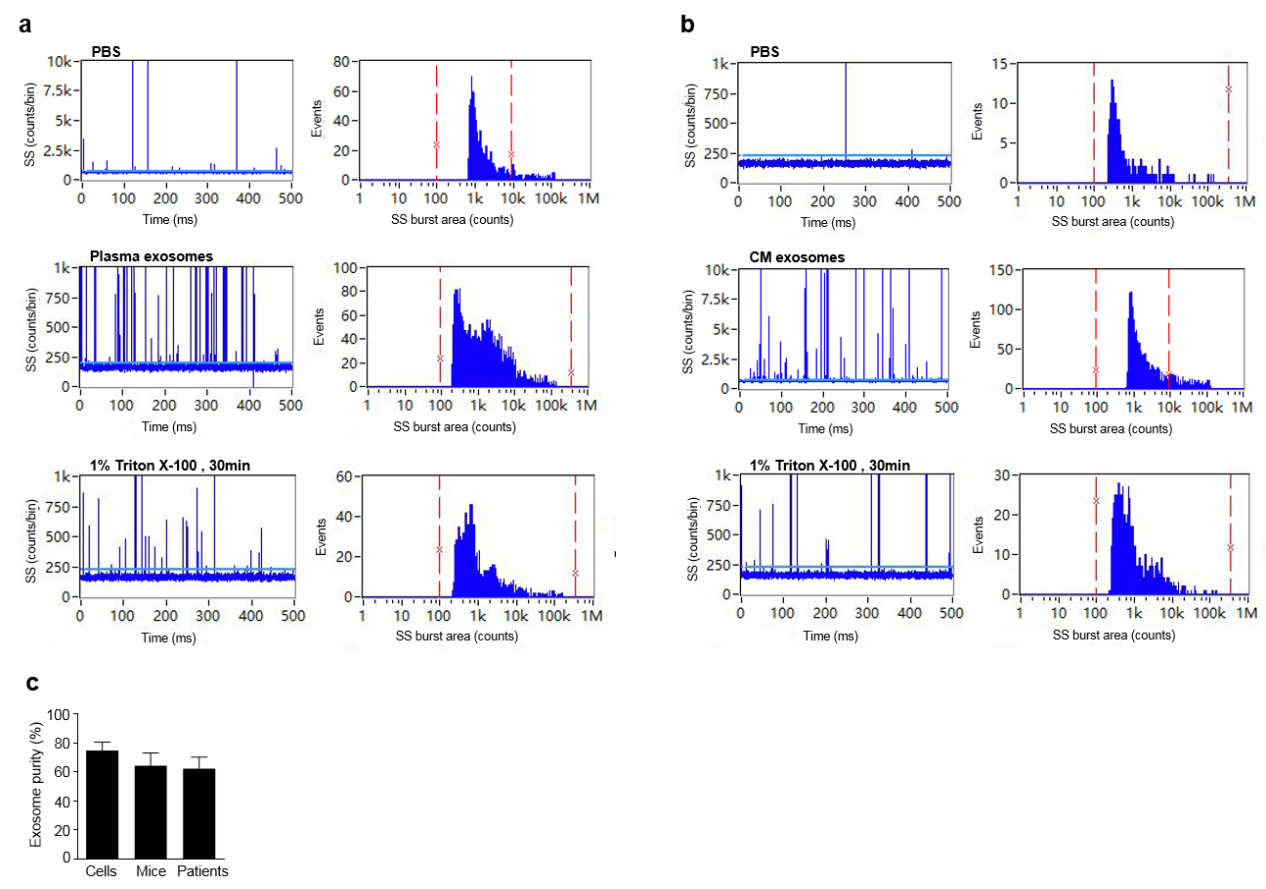
Fig. S7 Side scatter intensity distributions and purities of exosomes isolated both from plasma of the patients/mice and from culture media of the CT26/LLC cells**. (a), (b) Representative side scatter (SS) burst traces and intensity distributions of PBS and exosomes before and after treatment with 1% Triton X-100, which were isolated from plasma of the patients/mice (a), and culture media of the CT26/LLC cells (b). Intensity distributions of the exosomes were analyzed with a high-sensitivity Nano flow cytometer (HSFCM). (c) Purities of the isolated exosomes (n = 5).

**
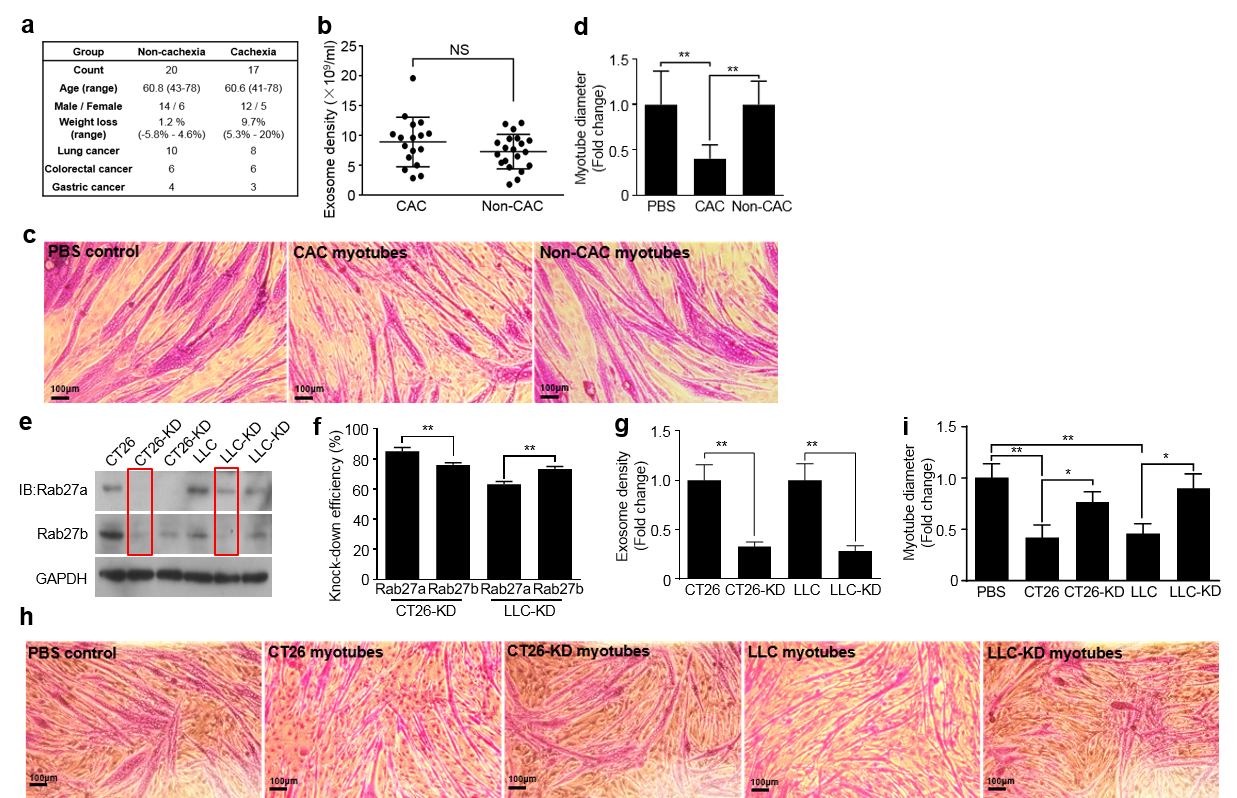
Fig. S8 Tumor-derived exosomes significantly induced myotube atrophy.** (a) Information of clinical cancer patients with or without cachexia symptoms (CAC patients and Non-CAC patients). (b) Plasma exosome densities of the CAC patients (n = 17) and the Non-CAC patients (n = 20). (c) Representative morphological appearances of myotubes incubated with either PBS or patient plasma-derived exosomes (scale bar = 100 *μ*m). (d) Diameters of the PBS controls, CAC and Non-CAC myotubes (n = 10). (e) Expressions of the Rab27a and Rab27b proteins in CT26, LLC, CT26-KD and LLC-KD cells (red boxes denote the knock-down cell lines used in the following experiments). (f) Quantification of the Rab27a and Rab27b knock-down efficiencies (n = 3). (g) Relative particle densities of exosomes isolated from conditioned media of CT26, LLC, CT26-KD, LLC-KD cells (n = 5). (h) Representative morphological appearances of CT26, LLC, CT26-KD and LLC-KD myotubes and PBS controls (Scale bar = 100 μm). (i) Relative diameters of CT26, LLC, CT26-KD and LLC-KD myotubes and PBS controls (n = 10). Statistically significances: *p* > 0.05, NS; *p* < 0.05, *; *p* < 0.01, **; *p* < 0.001, ***.


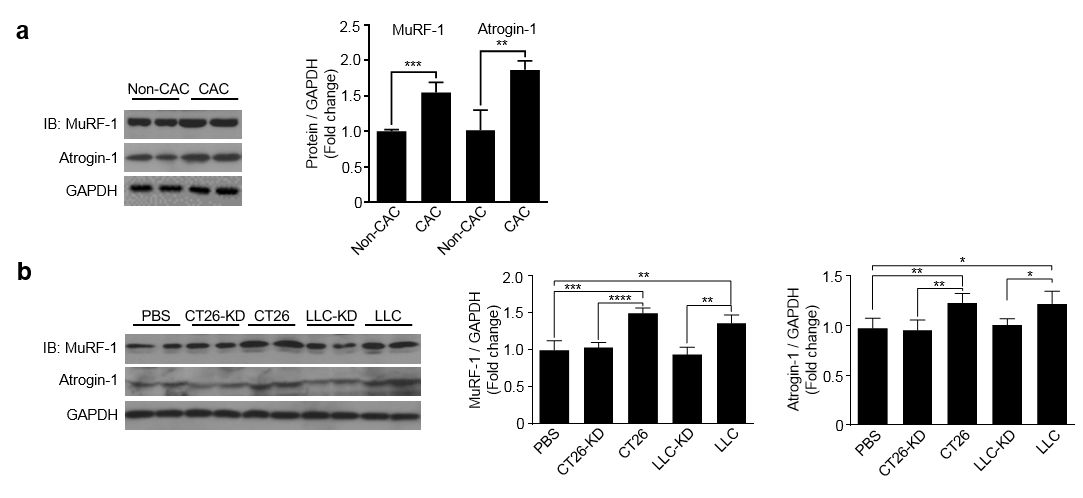


**Fig. S9 Expressions of MuRF-1 and Atrogin-1 proteins in the C2C12 myotubes**. (a) Myotubes incubated with plasma exosomes of the CAC and Non-CAC patients (n = 4). (b) Myotubes incubated with culture medium exosomes of the CT26, CT26-KD, LLC and LLC-KD cells (n = 4). Expressions of the MuRF-1 and Atrogin-1 proteins were analyzed by using western blot. Statistically significances: *p* > 0.05, NS; *p* < 0.05, *; *p* < 0.01, **; *p* < 0.001, ***.


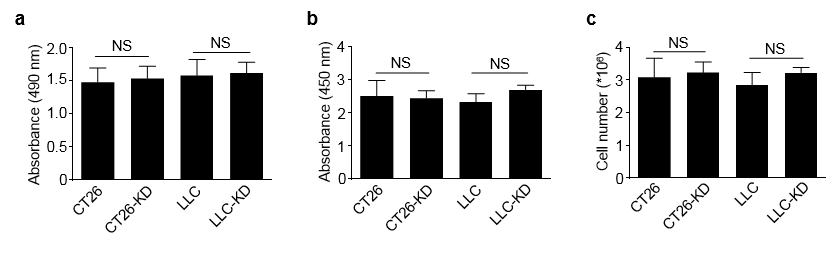


**Fig. S10** **Viabilities of the CT26, CT26-KD, LLC and LLC-KD tumor cells**. (a) MTS assay. (b) CCK-8 assay. (c) Cell count. n = 3 for each group. Statistically significances: *p* > 0.05, NS; *p* < 0.05, *; *p* < 0.01, **; *p* < 0.001, ***.

**
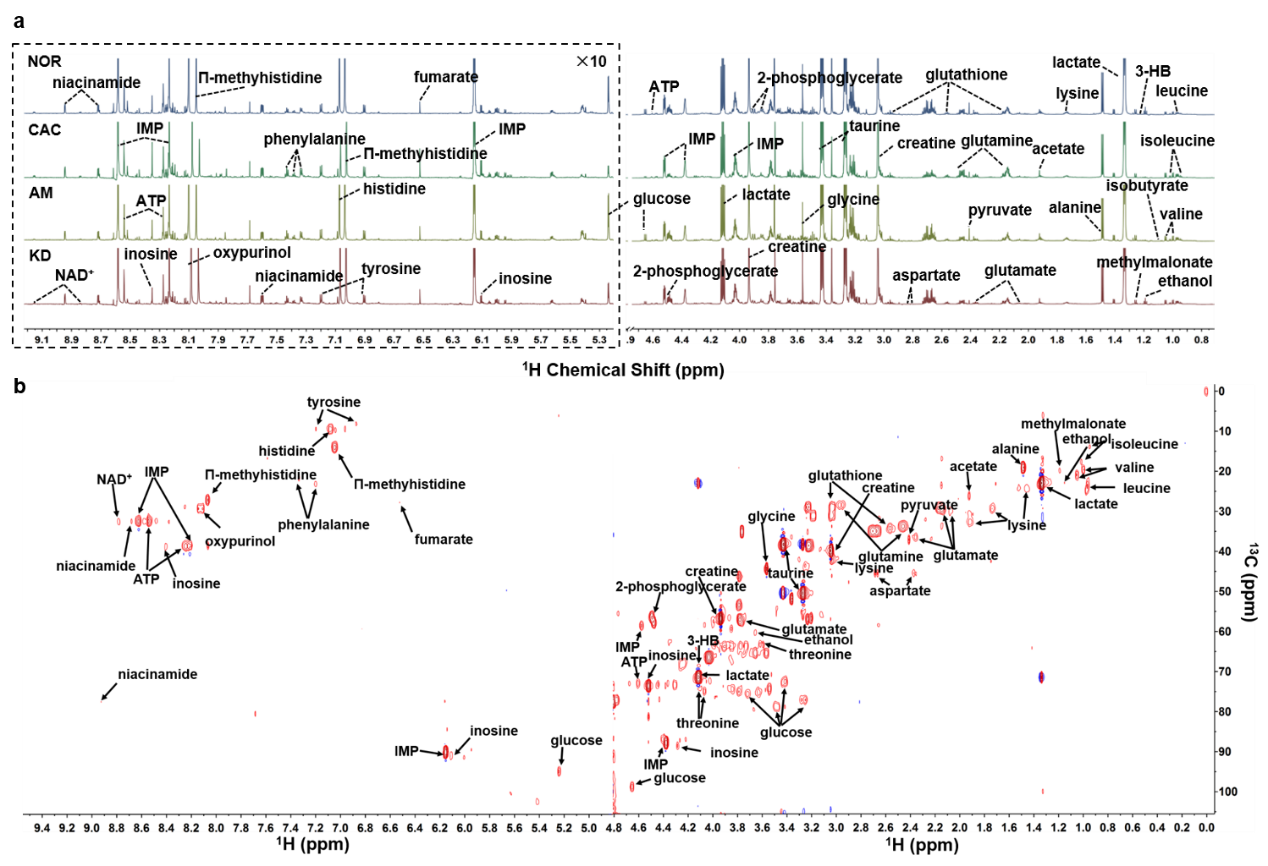
**

**Fig. S11 Typical 850 MHz NMR spectra recorded on aqueous extracts derived from the NOR, CAC, AM and KD gastrocnemius.** (a) Typical 1D ^1^H-NMR spectra of the four groups of mice. Vertical scales are kept constant in all the ^1^H spectra. Spectral regions of 0.5-4.7 ppm and 5.2-9.2 ppm are showed with removed water regions of 4.7-5.2 ppm. The region of 5.2-9.2 ppm has been magnified 10 times compared to another region of 0.5-4.7 ppm for clarity. Identified metabolites are shown in Supplementary Table S1. (b) Typical 2D ^1^H-^13^C HSQC spectrum of the mixture of aqueous extracts derived from cachectic gastrocnemius and normal control.


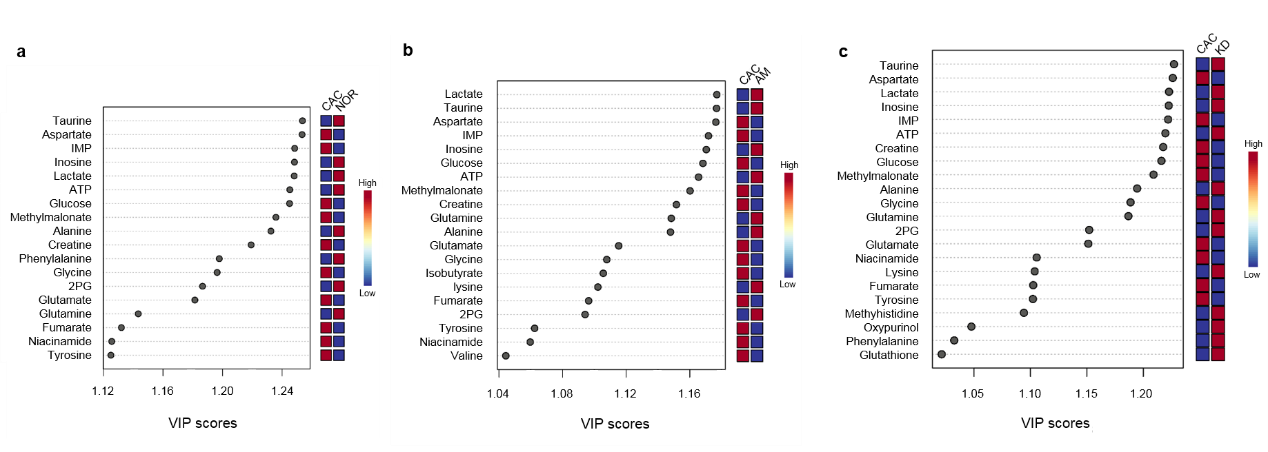


**Fig. S12 VIP score-ranking plots of significant metabolites identified from the PLS-DA models of the NOR, CAC, AM, KD gastrocnemius.** (a) CAC *vs.* NOR; (b) AM *vs.* CAC; (c) KD *vs.* CAC. The PLS-DA models were established based on relative integrals calculated from the 1D ^1^H-NMR spectra of aqueous gastrocnemius extracts. Significant metabolites were identified with VIP >1.

**
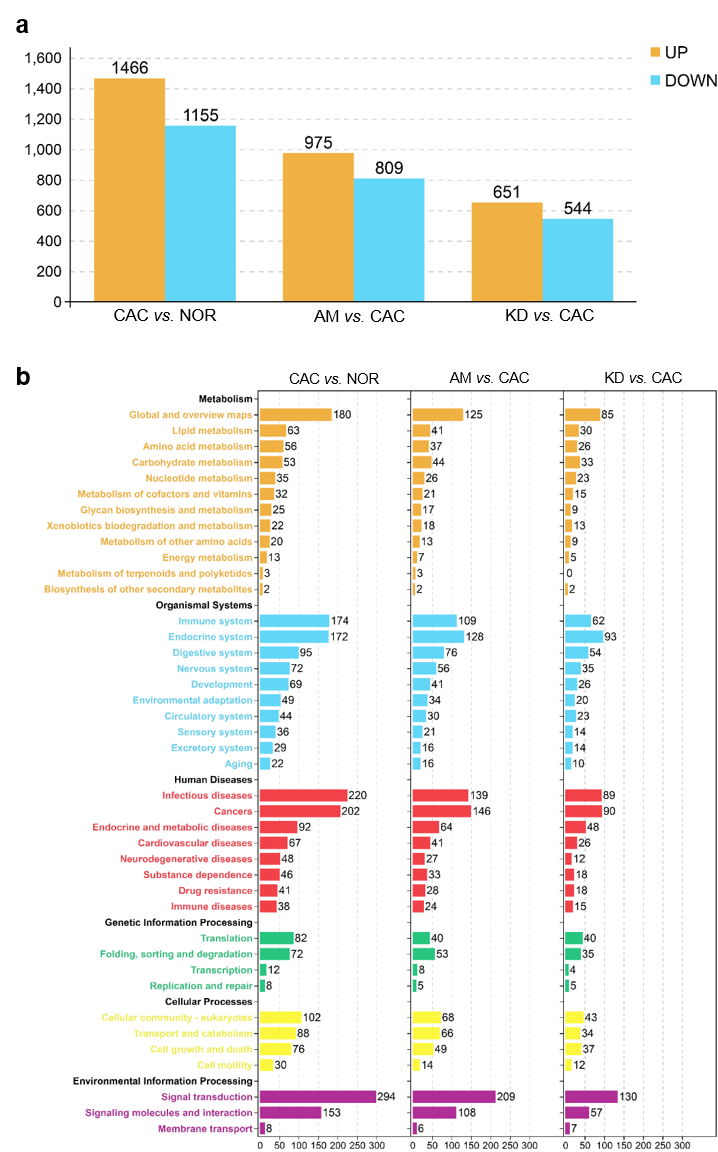
**

**Fig. S13 Transcriptomic profiling of** **the** **NOR, CAC, AM and KD gastrocnemius.** (a) Histogram illustrating numbers of differentially expressed genes (DEGs) identified from the pairwise comparisons of CAC *vs*. NOR, AM *vs*. CAC, KD *vs*. CAC (up/down-regulation for the comparison of A *vs.* B refers to A is up/down-regulated relative to B). (b) Significantly changed biological processes identified from the KEGG enrichment analysis.

**
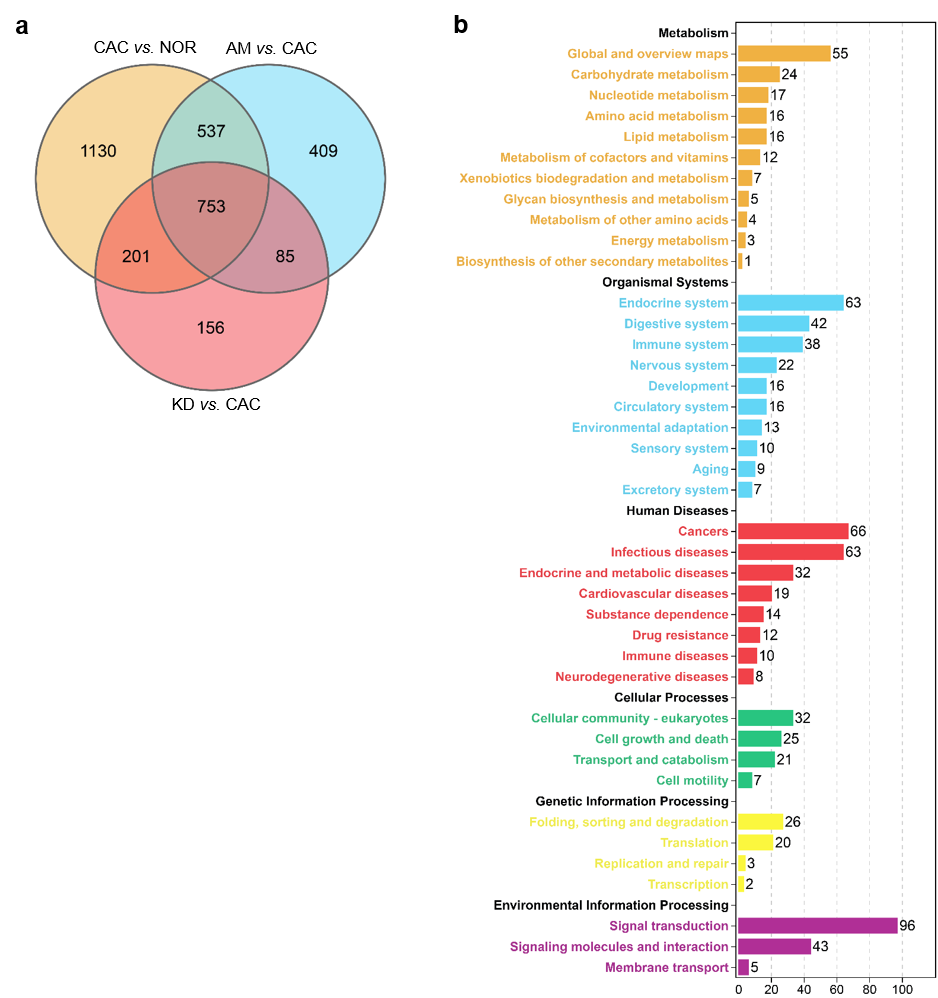
**

**Fig. S14 Numbers of differentially expressed genes shared by pairwise comparisons among the NOR, CAC, AM and KD gastrocnemius.** (a) Venn diagram illustrating the numbers of the DEGs shared by the pairwise comparisons of CAC *vs.* NOR, AM *vs.* CAC, KD *vs.* CAC. (b) Significantly changed biological processes identified from the KEGG enrichment analysis based on the DEGs shared simultaneously by the three pairwise comparisons (753 genes).


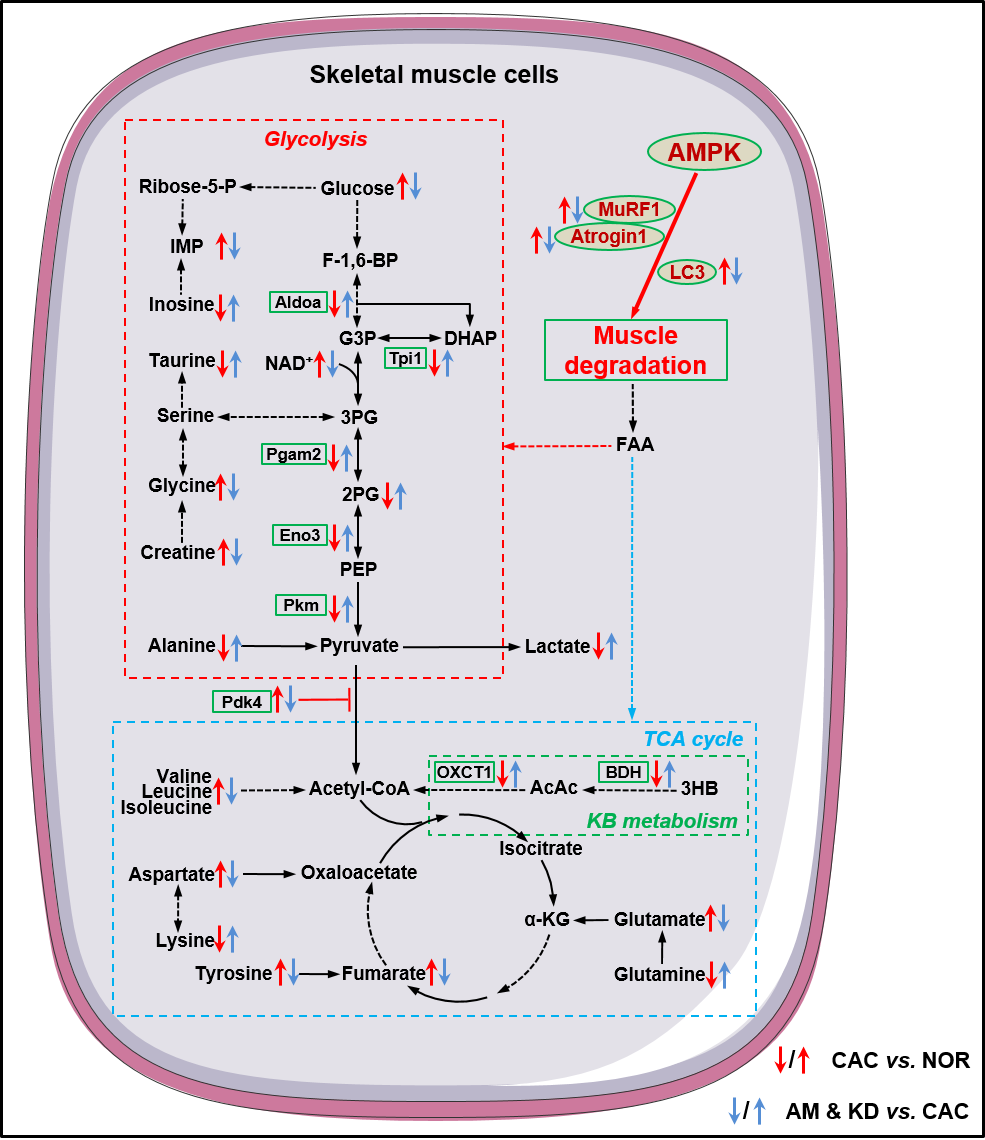


**Fig. S15** **Illustration of the effects of the amiloride treatment for improving metabolic impairments in cachectic gastrocnemius.** In cachectic muscles, AMPK activates muscular protein degradation, enhancing the levels of amino acids and acetyl-CoA which are further supplemented into glycolysis and TCA cycle for energy production. Both the blockage of glycolysis and the obstacle to ketone body utilization revealed by the integrated metabolomic and transcriptomic analyses, significantly contribute to cachectic muscle wasting. The amiloride treatment improves metabolic impairments in cachectic gastrocnemius and significantly ameliorate muscle wasting in cancer cachexia, thereby alleviating cancer cachexia progression. Abbreviations: NOR, normal control mice; CAC, CT26/LLC cachexia mice; AM: amiloride-treated mice; KD, mice inoculated with *Rab27* knockdown CT26/LLC cells; OXCT1, 3-oxoacid CoA transferase 1; BDH2, 3-hydroxybutyrate dehydrogenase 2; FFA, free fatty acid; FAA, free amino acid; KB, ketone body; α-KG, α-ketoglutarate, AcAc, acetoacetic acid; F-1,6-BP, fructose-1,6-bisphosphate; G3P, glyceraldehyde-3-phosphate; DHAP, Dihydroxyacetone phosphate; 3PG, 3-phosphoglycerate; 2PG, 2-phosphoglycerate; PEP, phosphoenolpyruvate; 3-HB, 3-hydroxybutyrate; IMP, inosine monophosphate.

**Table S1** **Information of the used antibodies and reagents.**

| **REAGENT or RESOURCE** | **SOURCE** | **IDENTIFIER** |
| --- | --- | --- |
| **Antibody** | | |
| MuRF1 | Abcam | ab172479 |
| Fbx32 | Abcam | ab168372 |
| MyoD1 | Abcam | ab64159 |
| Myogenin | Proteintech | 67082-1-lg |
| Myosin Light Chain 2 | Proteintech | 10906-1-AP |
| Myosin Ⅱb | Cell Signaling Technology | 3405S |
| AMPKα | Cell Signaling Technology | 2532S |
| Phospho-AMPKα (Thr172) | Cell Signaling Technology | 2535S |
| Akt | Proteintech | 10176-2-AP |
| Phospho-Akt(Thr308) | Cell Signaling Technology | 13038T |
| Phospho-Akt(Ser473) | ABGENT | AP3434A |
| STAT3 | Proteintech | 20253-2-AP |
| Phospho-Stat3 (Tyr705) | Cell Signaling Technology | 9145S |
| FOXO3A | Proteintech | 10849-1-AP |
| Phospho-FoxO3a(Ser253) | Cell Signaling Technology | 9466S |
| LC3 | Proteintech | 14600-1-AP |
| ACAT1 | Affinity biosciences | DF3726 |
| OXCT1 | Affinity biosciences | DF12082 |
| BDH2 | Proteintech | 27207-1-AP |
| CD36 | Proteintech | 18836-1-AP |
| MCT1 | Proteintech | 20139-1-AP |
| MCT4 | Proteintech | 22787-1-AP |
| ACOT1 | Abcam | Ab100915 |
| TSG101 | Proteintech | 14497-1-AP |
| HSP70 | Proteintech | 10995-1-AP |
| CD9 | Proteintech | 20597-1-AP |
| RAB27A | Proteintech | 17817-1-AP |
| RAB27B | Proteintech | 13412-1-AP |
| GAPDH | Proteintech | 10494-1-AP |
| **Commercial kit** | | |
| MTS | Promega Corporation | G5421 |
| CCK-8 | Beyotime | C0038 |
| ELISA mouse TNF-α | R&D SYSTEMS | MTA00B |
| ELISA mouse IL-6 | R&D SYSTEMS | M6000B |
| ELISA mouse IL-1β | R&D SYSTEMS | MLB00C |
| **Chemical** | | |
| Amiloride | Sigma-Aldrich | 1019701 |
| GW4869 | Sigma-Aldrich | D1692 |
| Turbofect | Thermo Fisher scientific | R0531 |
| Protease & Phosphotase Inhibitor | Thermo Fisher scientific | 78443 |

**Table S2** **Metabolites identified in ^1^H NMR spectra of aqueous extracts from gastrocnemius.**

| **NO.** | **Metabolite** | **δ ^1^H (ppm) and multiplicity** |
| --- | --- | --- |
| 1 | Leucine | 0.96(d), 0.97(d), 1.69(m), 1.70(m), 1.73(m), 3.73(m) |
| 2 | Isoleucine | 0.94(t), 1.01(d), 1.21(m), 1.42(m), 2.00(m), 3.67(d) |
| 3 | Valine | 0.99(d), 1.05(d), 2.26(m), 3.60(d) |
| 4 | Isobutyrate | 1.05(d), 2.37(m) |
| 5 | Ethanol | 1.17(t), 3.65(q) |
| 6 | 3-HB | 1.20(d), 2.30(q), 2.39(q), 4.14(m) |
| 7 | Methylmalonate | 1.25(d), 3.15(q) |
| 8 | Alanine | 1.47(d), 3.78(q) |
| 9 | Lysine | 1.43(m), 1.50(m), 1.73(m), 1.89(m), 1.92(m), 3.02(t), 3.75(t) |
| 10 | Acetate | 1.91(s) |
| 11 | Glutamate | 2.08(m), 2.12(m), 2.34(m), 2.37(m), 3.75(m) |
| 12 | Glutamine | 2.13(m), 2.45(m), 3.77(t) |
| 13 | Pyruvate | 2.41(s) |
| 14 | Glutathione | 2.15(m), 2.55(m), 2.96(m), 3.77(m), 4.56(m) |
| 15 | Aspartate | 2.68(dd); 2.81(dd); 3.90(dd) |
| 16 | Creatine | 3.04(s), 3.93(s) |
| 17 | Taurine | 3.24(t), 3.41(t) |
| 18 | Glycine | 3.57(s) |
| 19 | IMP | 4.02(m), 4.37(m), 4.52(q), 6.15(d), 8.23(s), 8.58(s) |
| 20 | Lactate | 1.33(d), 4.11(q) |
| 21 | 2-Phosphoglycerate | 3.81(q), 3.88(q), 4.49(m) |
| 22 | Glucose | β(3.24(dd), 3.48(t), 3.90(dd)), α(3.54(dd), 3.71(t), 3.72(dd), 3.83(m) |
| 23 | Inosine | 3.83(d), 3.84(d), 6.1(d), 8.23(s), 8.35(s) |
| 24 | Fumarate | 6.51(s) |
| 25 | Tyrosine | 3.05(dd), 3.19(dd), 6.92(d), 7.19(d) |
| 26 | Methyhistidine | 3.18(q), 3.92(q), 7.02(s), 7.84(s) |
| 27 | Histidine | 7.06(s), 7.85(s) |
| 28 | Phenylalanine | 3.12(dd), 3.30(dd), 3.99(dd), 7.33(d), 7.37(t),7.43(t) |
| 29 | Niacinamide | 7.60(q), 8.23(m), 8.71(m), 8.94(m) |
| 30 | Oxypurinol | 8.08(s) |
| 31 | ATP | 6.14(d), 8.27(s), 8.58(s) |
| 32 | NAD^+^ | 6.03(d), 6.08(s), 8.16(s), 8.20(m), 8.41(s), 8.82(d), 9.13(d), 9.32(s) |

Note: Multiplicity: s, singlet; d, double; t, triplet; q, quartet; m, multiple; dd, double of double.

**Table S3** **Quantitative comparison of metabolite levels based on relative integrals calculated from the 1D ^1^H NMR spectra of aqueous gastrocnemius extracts.**

| **Metabolite** | **Multiple comparisons** | | | **Mean ± SD** | | | | **One-way ANOVA** | |
| --- | --- | --- | --- | --- | --- | --- | --- | --- | --- |
|  | CAC *vs.* NOR | AM vs. CAC | KD  *vs.* CAC | NOR | CAC | AM | KD | F | P |
| Leucine | ↑↑ | ↓↓↓ | ↓↓ | 0.083±0.008 | 0.119±0.019 | 0.057±0.017 | 0.079±0.014 | 16.865 | ＜0.001 |
| Isoleucine | ↑↑ | ↓↓↓ | ↓↓ | 0.018±0.002 | 0.027±0.005 | 0.011±0.004 | 0.017±0.004 | 16.106 | ＜0.001 |
| Valine | ↑↑ | ↓↓↓ | ↓↓ | 0.034±0.003 | 0.050±0.009 | 0.022±0.006 | 0.033±0.006 | 17.976 | ＜0.001 |
| Isobutyrate | ↑ | ↓↓↓ | NS | 0.001±0.001 | 0.003±0.001 | 0.000±0.000 | 0.002±0.001 | 5.797 | 0.005 |
| Ethanol | NS | NS | NS | 0.008±0.002 | 0.007±0.004 | 0.008±0.007 | 0.005±0.005 | 0.77 | 0.524 |
| 3-HB | NS | NS | NS | 0.003±0.001 | 0.003±0.011 | 0.002±0.001 | 0.003±0.001 | 0.559 | 0.654 |
| Methylmalonate | ↑↑↑ | ↓↓↓ | ↓↓↓ | 0.032±0.002 | 0.478±0.118 | 0.029±0.004 | 0.031±0.005 | 86.217 | ＜0.001 |
| Alanine | ↓↓↓ | ↑↑↑ | ↑↑↑ | 0.323±0.068 | 0.140±0.068 | 0.277±0.045 | 0.291±0.048 | 14.98 | ＜0.001 |
| Lysine | ↓ | ↑↑ | ↑↑ | 0.150±0.056 | 0.076±0.020 | 0.130±0.032 | 0.135±0.025 | 4.831 | 0.011 |
| Acetate | NS | ↓ | NS | 0.043±0.010 | 0.058±0.013 | 0.040±0.011 | 0.053±0.016 | 2.454 | 0.093 |
| Glutamate | ↑↑↑ | ↓↓↓ | ↓↓↓ | 0.043±0.008 | 0.171±0.039 | 0.036±0.006 | 0.042±0.007 | 62.012 | ＜0.001 |
| Glutamine | ↓↓ | ↑↑↑ | ↑↑↑ | 0.108±0.031 | 0.029±0.011 | 0.099±0.016 | 0.091±0.017 | 19.006 | ＜0.001 |
| Pyruvate | ↓ | ↑↑ | NS | 0.018±0.004 | 0.013±0.004 | 0.025±0.007 | 0.018±0.009 | 3.92 | 0.026 |
| Glutathione | NS | NS | NS | 0.005±0.002 | 0.004±0.001 | 0.005±0.001 | 0.005±0.001 | 0.576 | 0.638 |
| Aspartate | ↑↑↑ | ↓↓↓ | ↓↓↓ | 0.002±0.001 | 3.136±0.372 | 0.001±0.001 | 0.001±0.001 | 426.23 | ＜0.001 |
| Creatine | ↑↑↑ | ↓↓↓ | ↓↓↓ | 2.354±0.390 | 4.308±0.520 | 2.177±0.229 | 2.172±0.229 | 49.149 | ＜0.001 |
| Taurine | ↓↓↓ | ↑↑↑ | ↑↑↑ | 2.887±0.404 | 0.291±0.090 | 2.773±0.393 | 2.879±0.253 | 100.56 | ＜0.001 |
| Glycine | ↑↑↑ | ↓↓↓ | ↓↓↓ | 0.180±0.039 | 0.400±0.035 | 0.143±0.033 | 0.126±0.030 | 80.991 | ＜0.001 |
| IMP | ↑↑↑ | ↓↓↓ | ↓↓↓ | 0.283±0.058 | 1.640±0.250 | 0.261±0.020 | 0.250±0.032 | 169.23 | ＜0.001 |
| Lactate | ↓↓↓ | ↑↑↑ | ↑↑↑ | 1.199±0.123 | 0.129±0.030 | 1.193±0.144 | 1.204±0.138 | 123.31 | ＜0.001 |
| 2-Phosphoglycerate | ↓↓↓ | ↑↑↑ | ↑↑↑ | 0.127±0.034 | 0.025±0.012 | 0.108±0.034 | 0.108±0.028 | 15.482 | ＜0.001 |
| Glucose | ↑↑↑ | ↓↓↓ | ↓↓↓ | 0.028±0.011 | 0.330±0.030 | 0.030±0.012 | 0.028±0.015 | 391.45 | ＜0.001 |
| Inosine | ↓↓↓ | ↑↑↑ | ↑↑↑ | 0.233±0.046 | 0.010±0.003 | 0.216±0.015 | 0.203±0.028 | 82.992 | ＜0.001 |
| Fumarate | ↑↑↑ | ↓↓↓ | ↓↓↓ | 0.004±0.002 | 0.015±0.003 | 0.003±0.001 | 0.004±0.002 | 51.966 | ＜0.001 |
| Tyrosine | ↑↑ | ↓↓ | ↓↓ | 0.010±0.001 | 0.122±0.046 | 0.007±0.001 | 0.009±0.003 | 35.721 | ＜0.001 |
| Methyhistidine | NS | NS | ↑ | 0.090±0.024 | 0.049±0.043 | 0.096±0.033 | 0.111±0.019 | 4.346 | 0.016 |
| Histidine | NS | NS | NS | 0.028±0.027 | 0.015±0.003 | 0.035±0.039 | 0.020±0.027 | 0.634 | 0.602 |
| Phenylalanine | NS | ↓ | NS | 0.012±0.001 | 0.011±0.002 | 0.008±0.002 | 0.012±0.003 | 4.822 | 0.011 |
| Niacinamide | ↑↑ | ↓↓ | ↓↓ | 0.008±0.002 | 0.085±0.039 | 0.007±0.001 | 0.007±0.001 | 23.262 | ＜0.001 |
| Oxypurinol | NS | ↑ | ↑↑ | 0.055±0.038 | 0.023±0.003 | 0.064±0.034 | 0.093±0.038 | 4.794 | 0.011 |
| ATP | ↓↓↓ | ↑↑↑ | ↑↑↑ | 0.016±0.003 | 0.002±0.001 | 0.015±0.001 | 0.014±0.002 | 75.411 | ＜0.001 |
| NAD^+^ | ↑↑ | ↓ | ↓↓ | 0.001±0.000 | 0.002±0.001 | 0.001±0.000 | 0.000±0.000 | 9.995 | ＜0.001 |

Note**:** Statistical significances were determined by one-way ANOVA analysis followed by Tukey’s multiple comparison test, and represented by the *p* values: NS, *p* > 0.05; ↓/↑, *p* < 0.05; ↓↓/↑↑, *p* < 0.01; ↓↓↓/↑↑↑, *p* < 0.001. Differential metabolites were identified with *p* < 0.05. The upward arrow and downward arrow denote that the difference between A and B is positive (A is increased compared to B) and negative (A is decreased compared to B), respectively.

**Table S4 Characteristic metabolites identified from pairwise comparisons of the four groups of gastrocnemius.**

| **Metabolite** | **CAC *vs.* NOR** | **AM *vs.* CAC** | **KD *vs.* CAC** |
| --- | --- | --- | --- |
| Glucose | ↑ | ↓ | ↓ |
| Lactate | ↓ | ↑ | ↑ |
| Taurine | ↓ | ↑ | ↑ |
| IMP | ↑ | ↓ | ↓ |
| Inosine | ↓ | ↑ | ↑ |
| Glycine | ↑ | ↓ | ↓ |
| Alanine | ↓ | ↑ | ↑ |
| 2PG | ↓ | ↑ | ↑ |
| Creatine | ↑ | ↓ | ↓ |
| Methylmalonate | ↑ | ↓ | ↓ |
| Niacinamide | ↑ | ↓ | ↓ |
| ATP | ↓ | ↑ | ↑ |
| Aspartate | ↑ | ↓ | ↓ |
| Glutamate | ↑ | ↓ | ↓ |
| Glutamine | ↓ | ↑ | ↑ |
| Fumarate | ↑ | ↓ | ↓ |
| Tyrosine | ↑ | ↓ | ↓ |
| Isobutyrate |  | ↑ |  |
| lysine |  | ↓ | ↓ |
| Valine |  | ↑ |  |
| Oxypurinol |  |  | ↓ |

Note: Characteristic metabolites were determined by a combination of the significant metabolites identified from the PLS-DA analyses (VIP > 1) and differential metabolites identified from the univariate analyses (*p* < 0.05). The upward arrow and downward arrow denote that the difference between A and B is positive (A is increased compared to B) and negative (A is decreased compared to B), respectively.

**Table S5** **Muscular atrophy-related differentially expressed genes (DEGs) identified from the pairwise comparison of cachectic gastrocnemius and normal control.**

| **Gene ID** | **log_2_(FC)** | ***p* Value** | **FDR** | **Symbol** | **Description** |
| --- | --- | --- | --- | --- | --- |
| ENSMUSG00000068614 | -5.253 | 7.086E-14 | 8.210E-12 | Actc1 | actin, alpha, cardiac muscle 1 |
| ENSMUSG00000059201 | -2.186 | 4.849E-02 | 1.621E-01 | Lep | leptin |
| ENSMUSG00000114515 | -2.067 | 6.771E-07 | 1.650E-05 | Aldoa | aldolase A, fructose-bisphosphate |
| ENSMUSG00000026185 | -1.968 | 3.060E-10 | 1.700E-08 | Igfbp5 | insulin-like growth factor binding protein 5 |
| ENSMUSG00000061816 | -1.711 | 2.074E-06 | 4.360E-05 | Myl1 | myosin, light polypeptide 1 |
| ENSMUSG00000060600 | -1.392 | 9.287E-08 | 2.910E-06 | Eno3 | enolase 3, beta muscle |
| ENSMUSG00000020475 | -1.343 | 1.498E-07 | 4.410E-06 | Pgam2 | phosphoglycerate mutase 2 |
| ENSMUSG00000023456 | -1.321 | 4.093E-09 | 1.740E-07 | Tpi1 | triosephosphate isomerase 1 |
| ENSMUSG00000032294 | -1.242 | 2.271E-09 | 1.030E-07 | Pkm | pyruvate kinase, muscle |
| ENSMUSG00000035923 | 1.332 | 6.032E-21 | 2.230E-18 | Myf6 | myogenic factor 6 |
| ENSMUSG00000078566 | 1.366 | 2.624E-17 | 5.900E-15 | Bnip3 | BCL2/adenovirus E1B interacting protein 3 |
| ENSMUSG00000025934 | 1.423 | 2.436E-02 | 9.793E-02 | Gsta3 | glutathione S-transferase, alpha 3 |
| ENSMUSG00000032202 | 1.520 | 1.558E-04 | 1.737E-03 | Rab27a | RAB27A, member RAS oncogene family |
| ENSMUSG00000027452 | 1.654 | 8.428E-13 | 7.980E-11 | Acss1 | acyl-CoA synthetase short-chain family member 1 |
| ENSMUSG00000019577 | 1.806 | 1.367E-03 | 1.018E-02 | Pdk4 | pyruvate dehydrogenase kinase, isoenzyme 4 |
| ENSMUSG00000038332 | 1.809 | 6.540E-13 | 6.270E-11 | Sesn1 | sestrin 1 |
| ENSMUSG00000072949 | 2.404 | 1.396E-16 | 2.780E-14 | Acot1 | acyl-CoA thioesterase 1 |
| ENSMUSG00000031490 | 2.547 | 8.691E-23 | 4.090E-20 | Eif4ebp1 | eukaryotic translation initiation factor 4E binding protein 1 |
| ENSMUSG00000028834 | 2.643 | 4.485E-25 | 3.480E-22 | Trim63 | tripartite motif-containing 63 |
| ENSMUSG00000022358 | 2.721 | 2.049E-24 | 1.330E-21 | Fbxo32 | F-box protein 32 |
| ENSMUSG00000071637 | 2.850 | 4.820E-14 | 5.960E-12 | Cebpd | CCAAT/enhancer binding protein (C/EBP), delta |
| ENSMUSG00000002769 | 2.903 | 2.936E-07 | 8.020E-06 | Gnmt | glycine N-methyltransferase |
| ENSMUSG00000023067 | 3.085 | 2.602E-14 | 3.340E-12 | Cdkn1a | cyclin-dependent kinase inhibitor 1A (P21) |
| ENSMUSG00000022094 | 2.039 | 4.110E-09 | 1.740E-07 | Slc39a14 | Cellular Processes, zinc transporter （ZIP14） |
| ENSMUSG00000004902 | 2.373 | 1.755E-02 | 7.606E-02 | Slc25a18 | solute carrier family 25 (mitochondrial carrier), member 18 |
| ENSMUSG00000032114 | -2.055 | 3.307E-18 | 8.560E-16 | Slc37a4 | solute carrier family 37 , member 4 |
| ENSMUSG00000002944 | 1.245 | 7.490E-05 | 3.547E-03 | Cd36 | CD36 molecule (FAT) |
| ENSMUSG00000022186 | -1.099 | 1.033E-04 | 4.547E-03 | Oxct1 | 3-oxoacid CoA transferase 1 |
| ENSMUSG00000028167 | -1.078 | 1.266E-01 | 5.578E-01 | Bdh2 | 3-hydroxybutyrate dehydrogenase, type 2 |
| ENSMUSG00000030739 | 1.167 | 3.510E-05 | 1.926E-03 | Myh14 | myosin, heavy polypeptide 14 |
| ENSMUSG00000031812 | 1.111 | 1.220E-05 | 7.968E-04 | Map1lc3b | microtubule-associated protein 1 light chain 3 beta |
| ENSMUSG00000027602 | 0.717 | 5.054E-03 | 8.513E-02 | Map1lc3a | microtubule-associated protein 1 light chain 3 alpha |
| ENSMUSG00000032508 | 1.058 | 6.055E-03 | 9.564E-02 | Myd88 | myeloid differentiation primary response gene 88 |

Note**:** The FC value refers to the fold change of the DEG expression level in the CAC gastrocnemius relative to the NOR gastrocnemius. DEGs were identified with two criteria: fold change of FC ≥ 1.5 or FC ≤ 0.67; false discovery rate (FDR) ≤ 0.05.
